# Understanding the Origins of Bacterial Resistance to Aminoglycosides through Molecular Dynamics Mutational Study of the Ribosomal A-Site

**DOI:** 10.1371/journal.pcbi.1002099

**Published:** 2011-07-21

**Authors:** Julia Romanowska, J. Andrew McCammon, Joanna Trylska

**Affiliations:** 1 Department of Biophysics, Faculty of Physics, University of Warsaw, Warsaw Poland; 2 Interdisciplinary Centre for Mathematical and Computational Modelling, University of Warsaw, Warsaw, Poland; 3 Department of Chemistry and Biochemistry, University of California, San Diego, California, United States of America; 4 Center for Theoretical Biological Physics, University of California, San Diego, California, United States of America; 5 Department of Pharmacology, University of California, San Diego, California, United States of America; 6 Howard Hughes Medical Institute, University of California, San Diego, California, United States of America; IBMC/CNRS, France

## Abstract

Paromomycin is an aminoglycosidic antibiotic that targets the RNA of the bacterial small ribosomal subunit. It binds in the A-site, which is one of the three tRNA binding sites, and affects translational fidelity by stabilizing two adenines (A1492 and A1493) in the flipped-out state. Experiments have shown that various mutations in the A-site result in bacterial resistance to aminoglycosides. In this study, we performed multiple molecular dynamics simulations of the mutated A-site RNA fragment in explicit solvent to analyze changes in the physicochemical features of the A-site that were introduced by substitutions of specific bases. The simulations were conducted for free RNA and in complex with paromomycin. We found that the specific mutations affect the shape and dynamics of the binding cleft as well as significantly alter its electrostatic properties. The most pronounced changes were observed in the U1406C∶U1495A mutant, where important hydrogen bonds between the RNA and paromomycin were disrupted. The present study aims to clarify the underlying physicochemical mechanisms of bacterial resistance to aminoglycosides due to target mutations.

## Author Summary

In hospitals throughout the world, aminoglycosidic antibiotics are used to combat even the most severe bacterial infections. However, the continuous emergence of resistant bacteria has created an urgent need to improve these antibiotics. Aminoglycosides bind to bacterial ribosomal RNA. Experiments have shown that specific point mutations in the RNA confer high resistance against aminoglycosides in bacteria. We performed molecular dynamics simulations of the aminoglycosidic binding site model after introducing various mutations. Here, we show that even single nucleotide substitutions can significantly change the physicochemical features of the binding site. In addition, we hypothesize why certain mutations result in bacterial resistance to aminoglycosides.

## Introduction

A well-known problem related to the use of antibacterial compounds is the emergence of resistant bacterial strains [1]. Bacteria constantly improve their resistance techniques by utilizing their abilities to mutate quickly. Their proliferation rate can be as short as minutes [2], and bacteria can also easily incorporate DNA from the environment. Therefore, there is a pressing need to identify new antibiotics that specifically and efficiently target the processes that are crucial for the life of the bacterial cell. One of the pivotal molecules in the cell is the ribosome, which is a macromolecular complex involved in peptide synthesis, and is composed of ribosomal RNA (rRNA) and proteins. The ribosome consists of two subunits: the small subunit (in prokaryotic organisms called 30S) and the large subunit (50S). Several antibiotics target various sites on the ribosomal subunits and interfere with bacterial translation at different stages.

Three transfer RNA (tRNA) binding sites are located at the interface between the 30S and 50S subunit (denoted as A, P, and E). The A-site on the 16S rRNA of the 30S subunit contains the binding site for most aminoglycosidic antibiotics [3], [4]. The nucleotide sequence of the A-site is highly conserved in all species [5], making it difficult for bacteria to gain resistance against aminoglycosides by simple random nucleotide substitutions, since mutations in these conservative regions often lead to death of bacterial cell [1], [6]. However, studies have shown that bacteria with only one mutation in the A-site, such as A1408G, which resembles the eukaryotic sequence, were no longer susceptible to aminoglycosides [7]–[9]. Furthermore, other experiments have proven that several other single point mutations exist that can successfully block the effect of these antibiotics [7],[10],[11]. However, aminoglycosides can bind to a variety of RNA targets and their specificity toward the A-site is not high. Therefore, finding out why a single base substitution in the A-site has such a large effect on the susceptibility of bacteria to aminoglycosides is of high relevance.

A variety of computational tools have emerged during the last few decades with the specific aim of complementing experimental structural approaches. In particular, molecular dynamics (MD) simulations have demonstrated the potential for revealing the dynamic and flexible properties of biomolecules at an atomic level of detail. Although the application of MD simulations to RNA is a relatively new field, much attention has been paid to adapt the MD methodology to these specific biomolecules (see refs. [12]–[14] for recent overviews of the improvements and achievements of the use of MD for nucleic acids). Several computational studies have been conducted on 16S rRNA fragments containing the A-site as well as on the entire ribosome. MD simulations – classical [15], replica-exchange [16] and targeted [17] – have shown that the adenines A1492 and A1493 are very mobile in the absence of the antibiotic. These bases are positioned opposite base 1408, and their mobility has been shown to be important for the fidelity of translation [17]–[22]. In the absence of the antibiotic, these adenines are almost in equilibrium between the flipped-out and flipped-in state, with a slight bias toward the flipped-in conformation [23], [24]. A1492 and A1493 are responsible for the proper recognition of tRNA, and upon the approach of the cognate tRNA, acquire an extra-helical position that accommodates the tRNA in the A-site. Aminoglycoside binding causes A1492 and A1493 to face to the outside of the 16S rRNA helix toward the solvent [25]–[28], which promotes the incorporation of near-cognate or non-cognate tRNAs. The MD studies mentioned above have shown that in the absence of antibiotic, the intra-helical state of A1492 and A1493 is energetically favored. Other MD simulations of the model A-site RNA fragment in complex with paromomycin [29], as well as with other aminoglycosides [30], have focused on the RNA solvation patterns and antibiotic binding free energies. Brownian dynamics simulation of the model A-site [31] and the entire 30S subunit [32] have investigated aminoglycoside association pathways and rates, but have not focused on the intrinsic dynamics of the binding site. Moreover, none of the theoretical studies to date have investigated the properties of the mutant A-site structures.

In our previous study [33], we identified the differences in physicochemical properties and internal dynamics of the model A-site between the prokaryotic and the eukaryotic-resembling structure when the adenine at position 1408 was substituted with guanine. In that study, we showed that the A1408G mutation affected the mobility of A1492 and A1493. We also observed that in the intra-helical state, these adenines sometimes form hydrogen bonds with the opposite base at position 1408. The base pair that formed is more stable in the eukaryotic-like structure (when guanine occupies position 1408) than in the prokaryotic structure (with adenine in position 1408). Most likely, the increased stability of this base pair has some hindrance to the binding of aminoglycosides to the A-site of the eukaryotic ribosome. We also observed that the A1408G substitution changes the electrostatic potential inside the binding cleft. Aminoglycosides are ionized in physiological pH [25], [34], and therefore electrostatic interactions are important for their proper binding.

Here, we have significantly extended our previous studies by analyzing how other experimentally reported mutations affect the features of the A-site RNA. We present the results of eight, 20 ns-long MD simulations of the model A-site mutated *in silico*: three single point mutants and one double mutant, both in the presence and absence of an aminoglycoside, paromomycin. The mutated sites were selected based on previous experimental studies [7], [10], [11], [35], where the authors compared the impact of different mutations in the A-site of *Mycobacterium smegmatis*, *Escherichia coli*, and other bacteria. The base substitutions that caused the most pronounced changes in minimal inhibitory concentrations (MIC) for selected bacterial species were chosen, especially in response to treatment with paromomycin.

## Results

**Figure 1:**
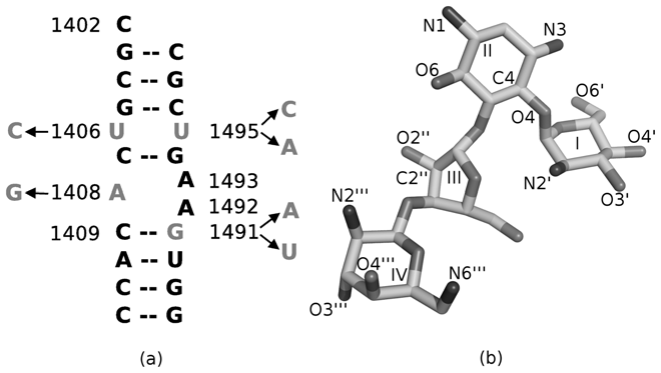
The binding site of paromomycin. (a) The sequence of the paromomycin binding site in the 16S ribosomal RNA (E. coli numbering); the mutated bases in our MD study are colored light gray and the arrows depict the applied mutations; the A1408G substitution accounts for the most important difference between the prokaryotic (A) and eukaryotic (G) sequence of the A-site and was previously analyzed [33]. The complete simulated structure contains two symmetric binding sites (A-sites) as in the crystal structure (PDB entry 1J7T). (b) A stick model of paromomycin heavy atoms showing atom names and ring numbering.

To investigate the influence of specific mutations on the physicochemical features of the 16S rRNA A-site, we performed MD simulations on a total of four mutated model A-site RNA, with and without the aminoglycoside representative, paromomycin (see Figure 1 and Table 1 for an overview of the substitutions). The crystal structure comprises two highly analogous A-sites, which only differ slightly in their atomic 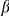-factor values. Therefore, all MD simulations and analyses were performed for both sections of the molecule in order to obtain better statistics of the observed properties and an increased sampling of the phase space.

**Table 1:** Summary of MD simulations.

*structure*		
*with (paromomycin)*	*without*	*effect of introducing mutation*
**NON_MUT_PAR**	**NON_MUT**	–
**G1491A_PAR**	**G1491A**	A is found in eukaryotic sequence [5], [39] and confers resistance against paromomycin (up to 64-fold increase in MIC values [10], [74])
**G1491U_PAR**	**G1491U**	high resistance against paromomycin (512-fold increase in MIC [7])
**U1495C_PAR**	**U1495C**	resistance against paromomycin (128-fold increase in MIC in *M. smegmatis* [7] and 5-fold increase in *T. thermophilus* [10])
**U1406C/U1495A_PAR**	**U1406C/U1495A**	high resistance against many aminoglycosides ( 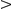 1000-fold increase in MIC for paromomycin [7], [75])

Labeling of MD simulations used in the text and the effects of introducing the mutations. All simulations were performed with 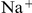 and 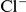 ions (for details see the *Methods* section).

As a comparative reference we performed an additional MD simulation of the wild-type bacterial sequence with bound paromomycin (denoted NON_MUT_PAR). This reference simulation preserved the majority of the bonds observed in the crystal structure (Figure S1) and the bound paromomycin retained its original conformation (root mean square fluctuations, RMSF, was less than 0.9 Å; discussed below). The behavior of the wild type bacterial A-site model without bound paromomycin was described in our previous work [33], which we also refer to throughout this study.

### Structural fluctuations of the A-site

**Figure 2:**
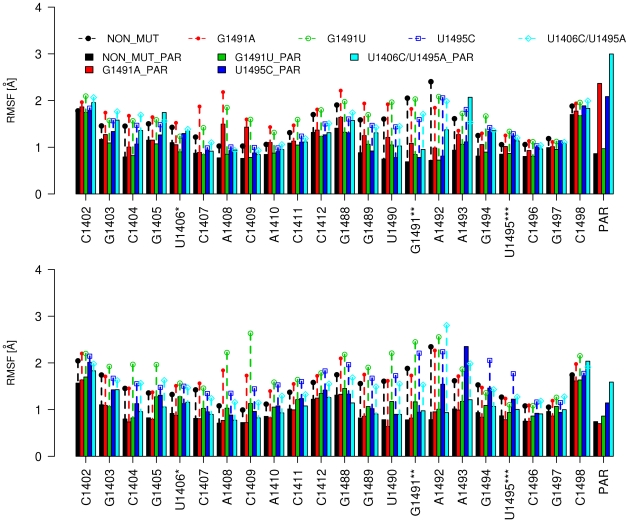
RMSF [Ångstrom] per residue. PAR denotes paromomycin; (*) the base is cytosine (C) in U1406C/U1495A and U1406C/U1495A_PAR; (**) the base is adenine (A) in G1491A and G1491A_PAR, and uracil (U) in G1491U and G1491U_PAR; (***) this base is adenine (A) in U1406C/U1495A and U1406C/U1495A_PAR, and cytosine (C) in U1495C and U1495C_PAR. The plot also shows the RMSF for the original, prokaryotic A-site structure [33]. Two graphs for each simulation depict RMSF of two symmetric fragments of the structure.

We analyzed the flexibility of the entire model A-site by calculating the average root mean square deviation (RMSD) of atomic positions and root mean square fluctuations (RMSF) of each nucleotide as well as paromomycin. The average RMSD from the initial structure, that was calculated for all heavy atoms, did not exceed 2.9 Å in every simulation (Figure S2). Previous studies have shown that paromomycin stabilizes both the wild-type [30], [33] and the A1408G mutated [33] A-site RNA structure (for base numbering see Figure 1a; throughout the paper the *E. coli* numbering convention of the A-site is used). In this study, we observed a similar stabilizing effect by the presence of paromomycin on the single point mutant structures G1491U and G1491A. A slightly less pronounced effect of paromomycin was also observed in the U1495C simulation. The stabilizing effects are reflected by the RMSF and RMSD values, which are shown in Figure 2 and Figure S2, respectively. For example, in the structure that contains the G1491U mutation without the drug, the bases that were in proximity to the mutated site as well as on the opposite strand of the RNA helix (i.e., A1408 and C1409) showed larger fluctuations, particularly in one section of the RNA fragment. In the presence of the antibiotic, all of the residues became more conformationally restrained.

In contrast, the overall decrease in RMSF (Figure 2) or RMSD (Figure S2) that occurred in the presence of the antibiotic was substantially less in the simulation with the double mutation (U1406C/U1495A vs. U1406C/U1495A_PAR). Unlike the other simulations of RNA with paromomycin, the U1406C/U1495A_PAR trajectory showed that the drug itself was more dynamic and significantly changed its conformation (RMSF values for the two paromomycin molecules in the structure: 2.99 and 1.59 Å). In addition, one paromomycin in the G1491A_PAR simulation was characterized by a higher RMSF of 2.8 Å, which indicated a change in conformation. This finding was confirmed by visualizing the trajectory (discussed below). The elevated RMSF of A1492, A1493, and A1408 were expected, since these three bases form a bulge in the original crystal structure and their flexibility is necessary for the fidelity of the translation process [17]–[22].

### Mutations change the mobility of A1492 and A1493

In the MD simulations of the original crystal structure of the model A-site without paromomycin [33] the adenines A1492 and A1493 were flexible and acquired both extra and intra-helical states. They moved from the flipped-out state to the flipped-in conformation, through the minor groove of the RNA helix.

Three important conformations of A1492 and A1493 can be distinguished [17], [19]–[22], [25]–[28]. Conformation (a), where both adenines occupy the inside of the RNA helix (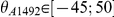, 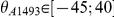; see “Methods” for the definition of the 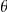 angle), which is a conformation that prevents the binding of the aminoglycoside and may also cause rejection of a non- or near-cognate tRNA during the translation process. In conformation (b) A1492 is flipped out (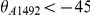 or 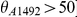) and A1493 stays inside the helix (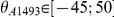); this conformation occurs when the translation termination factor has to be recognized and accepted. Finally, conformation (c), where both A1492 and A1493 are outside the RNA helix (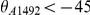 or 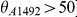, and 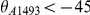 or 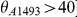), which occurs upon the acceptance of a cognate tRNA and also enables aminoglycoside binding.

**Figure 3:**
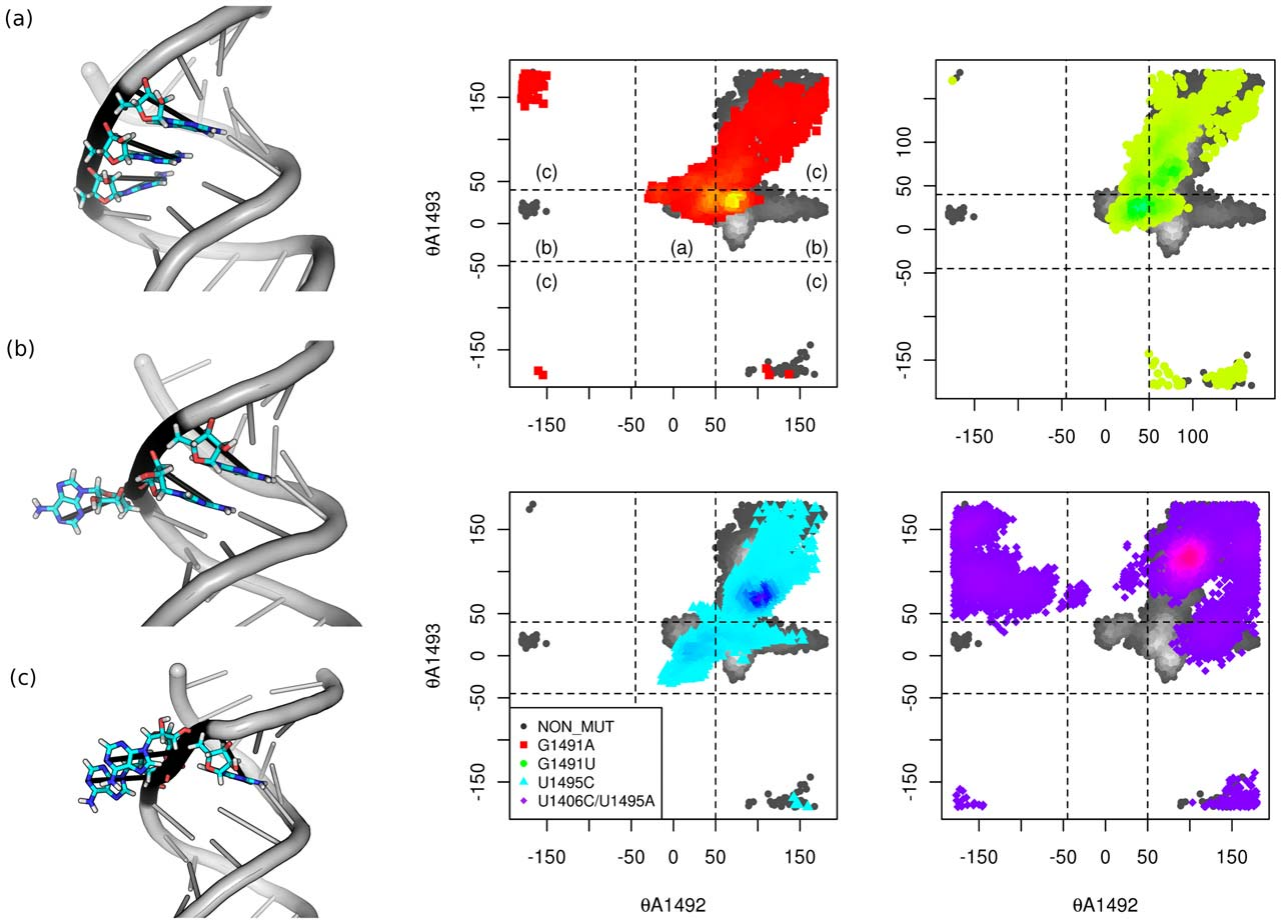
Mobility of A1492 and A1493. The pseudo-dihedral angle distribution showing the relative positioning of A1492 and A1493 with respect to A1494. Each plot presents the angle values sampled in both parts of the model A-site with the color variance indicating the sampling density. The panels on the left depict the three most important conformations (see text for the detailed description). The definition of the 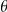 angle is presented in Figure S3 and described in Methods.

To quantify the variance of the conformations of A1492 and A1493 acquired in MD simulations, we used the pseudo-dihedral angle (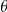) between the conformationally stable base G1494 and each of the adenines (Figure S3). From the distribution of the measured values (Figure 3), the changes in adenine motions caused by the mutations were observed. In addition, we calculated the overall percentages of time that the adenines were inside the RNA helix as another measurement of adenine flexibility (Table 2).

**Table 2:** Flipped-in conformation of A1492 and A1493.

	G1491A		G1491U		U1495C		U1406C/U1495A		NON_MUT^1^	
A1492	65.40	45.92	74.21	55.03	0.22	41.89	2.87	0.79	25.67	19.91
A1493	86.52	79.21	78.46	51.33	18.60	57.99	0.10	21.21	60.58	79.22

Percentage of simulation time when A1492 or A1493 was inside the helix (as defined by the pseudo-dihedral angle pictured in Figure S3 and described in the Methods section).

1 data from our previous study, see Ref. [33].

All of the conformations of A1492 and A1493 described above were observed in the NON_MUT simulations (Figure 3, dots in shades of gray). The G1491A and G1491U mutations restricted the adenines to the flipped-in ensemble of states (a). The smallest changes in the adenines' movement were introduced by the mutation U1495C, while the largest deviation from the original NON_MUT simulation was seen in the U1406C/U1495A simulation, where A1492 and A1493 were positioned outside of the helix for the majority of the time (Table 2).

Experimental studies have shown that the G1491A and G1491U mutations cause an increased read-through of the stop codon [36]. Based on the data presented in Figure 3, we noticed that the (b) area was almost not visited by the adenines in the mutated structures – they move as a pair, while the termination factor requires that only A1492 is in the flipped-out state [20], [21]. This may cause an acceptance of a non-cognate tRNA in place of a termination factor and lead to the read-through of a stop codon.

### Conformations of A1492 and A1493 influence the shape of the binding cleft

**Figure 4:**
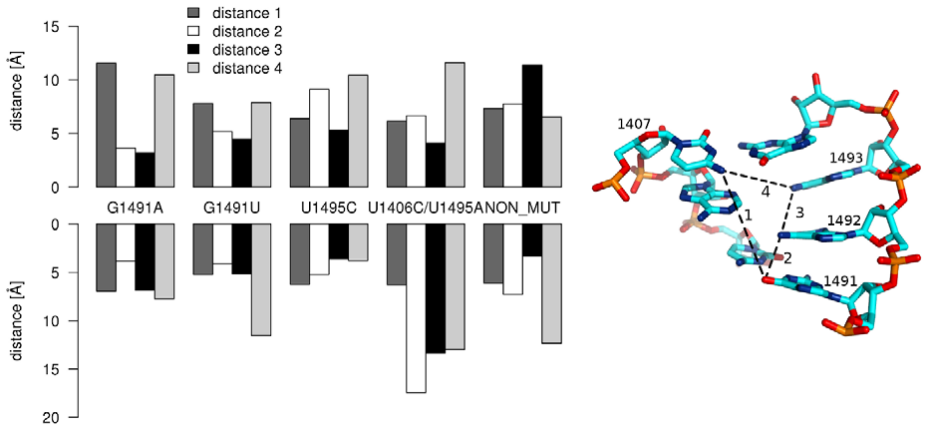
Distances inside the binding site in the most representative cluster. The inset presents the measured distances marked by dotted black lines.

Visualization of the G1491A and G1491U trajectories showed that the changes in base pairing and in the conformations of A1492 and A1493 made the A-site more condensed and compressed. We quantified these observations by calculating the distances between four atoms of residues 1407, 1491, 1492, and 1493 that pointed to the inside of the binding cleft. To simplify the presentation of these results, we have grouped the trajectory conformations into five clusters (see Methods, Table S1 and Figure S4). Figure 4 shows the distances between these four atoms in the representative structure (i.e., the structure that comprises the center of the cluster) of the most populated cluster.

In general, the cleft in the simulations with the mutated 1491 base was more compact than in the NON_MUT, even though bases A1492 and A1493 moved to the flipped-in state in all three simulations (NON_MUT, G1491A, and G1491U). We noticed that the change of the cleft shape was caused by the shift of the base pairing and the twisting of base 1491 (see visualization in Figure S4). Although distances 1 and 4 were large in one section of the RNA structure that was in the most populated cluster of the G1491A simulation (Figure 4 and Figure S4b, left), the data derived from the entire trajectory for both A-sites show that the mutations of G1491 resulted in the same or smaller dimensions of the binding site compared to the non-mutated structure (Figure S5).

Mutation of G1491 to adenine (G1491A) and to uracil (G1491U) allowed A1492 and A1493 to occupy the flipped-in state for up to 87% of the simulation time (Table 2). Therefore, the range of movement of A1492 and A1493 was reduced in these mutants (Figure S6). Especially the movement of the adenines in one of the A-sites of the G1491A mutant structure was more restricted to the flipped-in state when compared to the NON_MUT simulation (see also Figure 2, top). According to recent studies [17], [37], the decrease in movability is associated with a change in the accuracy of translation. In this case, the predominantly constant flipped-in position of A1492 and A1493 could result in a reduction in the number of cognate tRNAs accepted. Therefore, protein synthesis would be more prone to errors. On the other hand, it has been postulated that antibiotic binding occurs in a stochastic gating fashion [38], and thus a mutated A-site should be more resistant to aminoglycosidic antibiotics, since the drug would have difficulty in “catching” the A-site in a conformation that had flipped-out adenines. A recent experimental study [39] on the reverse mutation in the *yeast* ribosome (i.e., with the A1491G mutation) showed an analogous effect. The eukaryotic ribosome possessing a guanine in the 1491 position was less resistant to aminoglycosides. Moreover, there was a reduction in the frequency of translation error in the absence of the drug.

In contrast, mutation of the U·U pseudo-pair (i.e., in the simulations U1495C and U1406C/U1495A) caused A1492 and A1493 to occupy the outside of the RNA helix for the majority of the simulation time (Table 2 and Figure S6).

### Substitutions of G1491 destabilize base pairing

**Figure 5:**
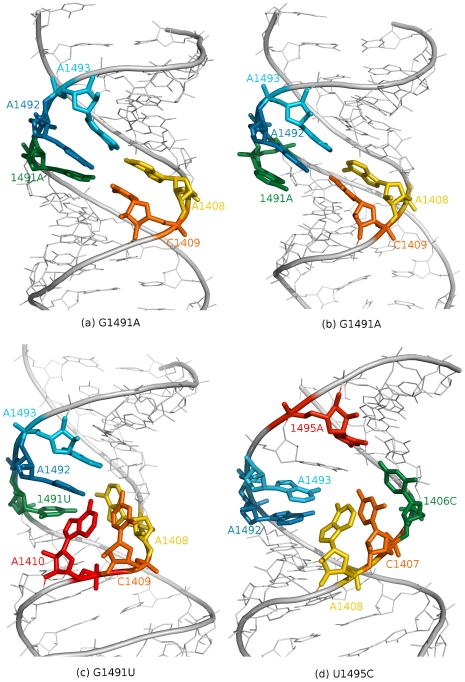
Trajectory snapshots. (a)–(c) The shift of base pairing observed in both simulations with the G1491 mutation. The pair formed with the mutated base was unstable. (d) The flipped-out conformation of the A1408 base, observed for a limited time in the U1495C simulation, showing the movability of bases in the bulge influenced by the U1495 mutation.

We observed that mutations in the G1491 position resulted in a change of the base pairing pattern near the substituted base. In the starting conformation, base 1491 formed a hydrogen bond with the opposite base C1409 (Figure 5a). These hydrogen bonds break several times in the G1491A and G1491U simulations. As a result, the C1409 base either pairs with A1492 (Figure 5b) or occupies the flipped-out state (Figure 5c). This type of shift in base pairing is commonly found in tertiary RNA structures [40], and it may contribute to bacterial resistance by changing the shape and volume of the binding site. A similar effect was also observed in some of our previous simulations of the wild-type prokaryotic A-site RNA fragment (for details see Ref. [33]); however, that shift was caused by a loss of stability by U1406·U1495, which prevented the flipped-in conformations of bases A1492 and A1493. In contrast, these two adenines were positioned inside the helix for the majority of the simulation time in the G1491A and G1491U simulations (Table 2).

The 1491U∶C1409 and 1491A∶C1409 pairs, which contained mutant G1491, were dynamic whenever they formed, and at times the C1409 base flipped out of the helix where it was stacked with either A1408 or A1410 (Figure 5c). Nevertheless, the G1491A mutant structure was generally more conformationally stable than the G1491U mutant. The base pair formed in the MD simulation with adenine in the 1491 position lasted approximately two times longer than with uracil in the same position (Table S2).

### The U·U pair loses its stability upon double mutation

The U1406·U1495 pair (Figure 1a) is important for the structural stability of the A-site and for proper distribution of electrostatic potential inside the cleft [7], [29]. Bound paromomycin forms one direct and one indirect hydrogen bond with the O4 oxygens of both uracils. Therefore, we monitored the behavior of these uridines in MD simulations to assess whether the mutations influence the base pairing and contacts with the drug.

**Figure 6:**
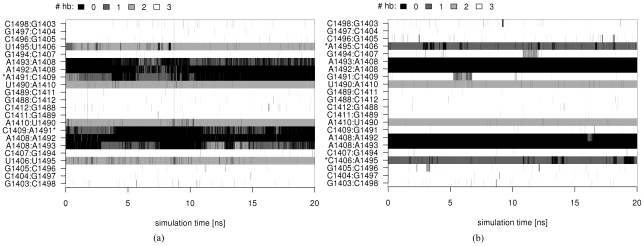
The number of hydrogen bonds formed between subsequent base pairs plotted versus simulation time. Data from simulations: (a) G1491A and (b) U1406C/U1495A. Asterisks (*) indicate the mutated bases.

**Figure 7:**
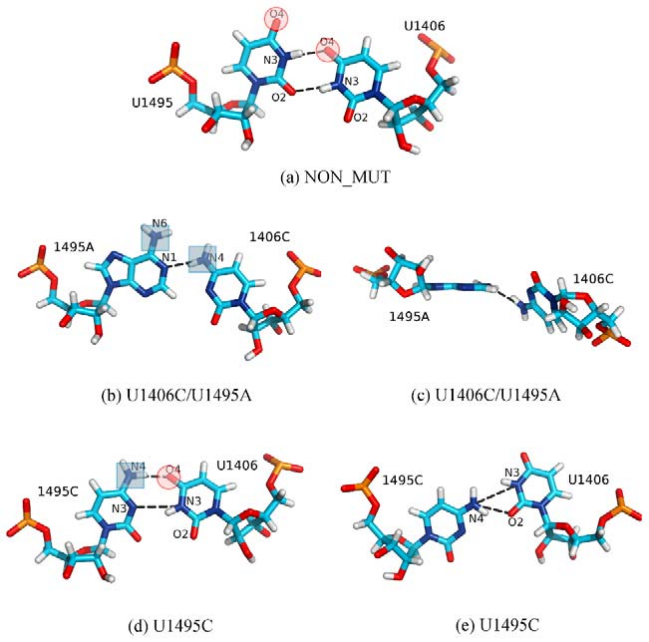
Mutations of the U1406·U1495 pair. Exemplary trajectory snapshots showing the interactions of the uracil pair before (a) and after introducing mutations (b)–(e). Pairs of sub-figures: (b) and (c), and (d) and (e) show different conformations of the base pair that were observed in the simulations. Notice the change in the charge carried by the mutated atoms: from negative charge (marked with red circles) to positive (marked with blue squares).

We found that the single mutants G1491U and G1491A did not affect the stability of the U1406·U1495 pair and that the pair was predominantly formed by two hydrogen bonds (Figure 6a and Table S2). In contrast, the resulting 1406C∶1495A pair from the U1406C/U1495A simulation formed one hydrogen bond and was only moderately stable (Figures 6b, 7b, and Table S2). Occasionally, 1406C was observed rotating to a position that was almost perpendicular to the base pair plane (Figure 7c). Nevertheless, the 1406C∶1495A pair often adopted an experimentally observed pattern(http://bps.rutgers.edu/atlas/bppattern/ac_5 [41]; Figure 7b).

The geometry and the partial charge distribution in this region were completely altered in the double mutant. Figures 7a, 7b, and 7d show the difference in the atom types and their positions in the original U1406·U1495 pair after introduction of the mutations. In the original U·U pair, two oxygens, U1406(O4) and U1495(O4), which were positioned inside the helix (Figure 7a), formed one direct and one indirect hydrogen bond with the neamine core of paromomycin (Rings I and II; Figure 1b). This moiety is present in every aminoglycoside and serves as an anchor for positioning the aminoglycosides in the A-site. By mutating the U·U pair to C∶A, the negative charge in the binding site that was provided by the uracil oxygens is deleted, which prevents the formation of important hydrogen bonds between the A-site and aminoglycosides. In addition, steric interactions can hinder the binding of the drug, since adenine is larger than uracil and occupies more space inside the binding cleft. The geometry-related changes could have been deduced from simple static structural modeling, however MD simulations describe the complicated dynamics of the hydrogen bonds that are formed between the nucleotides and between RNA and paromomycin. Mutating both uracils broke the U·U hydrogen bonding pattern and the adenines A1492 and A1493 were not able to adopt the flipped-in conformation for a longer period of time, making it easier for the aminoglycosides to bind to thus changed site.

In the U1495C simulation, where only one of the uracils was mutated to cytosine, the newly formed U∶C pair was conformationally stable (Table S2), and at times even formed three hydrogen bonds. The U∶C pair adopted a well-known pattern, called 4-carbonyl-amino [42] or *cis* W.C./W.C. [43] (Figure 7d), although alternate periods of a transient, non-classical conformation were also observed (Figure 7e). The mutated pair lacked one oxygen on the inner side of the base pair plane, and the uracil was often found situated deeper inside the helix than in the wild-type U·U conformation. This positioning of uracil may also make it more difficult for aminoglycosides to bind to a modified conformation.

### Double mutation of the U·U pair destabilizes bound paromomycin

**Figure 8:**
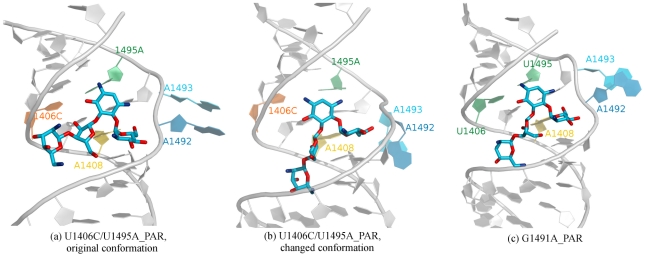
Conformations of paromomycin in the A-site. Exemplary trajectory snapshots showing the change of the conformation of paromomycin (compare (a) with (b) and (c)). Hydrogen atoms of paromomycin are not shown for clarity of the image.

The visualization of trajectory revealed changes in the internal dynamics of the A-site/paromomycin complex, which was a result of mutations of the U1406·U1495 pair in the U1406C/U1495A_PAR simulation. These changes were also observed in the RMSF (Figure 2). Due to the mutations, paromomycin changed its conformation (Figures 8a and 8b). Rings III and IV form the “tail” of paromomycin (Figure 1b) and are generally more mobile than the rest of the drug [32], [44]. However, in the U1406C/U1495A_PAR simulation, the centers of mass of rings III and IV shifted as much as 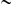
3 Å (Figure S8). In one of the A-sites, PAR(N2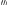) of ring IV formed a new hydrogen bond with the G1489(O2P). In the second A-site, a new hydrogen bond was formed between PAR(O3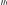) of ring IV and the 1406C(O2P). These were not observed for any other simulation.

**Figure 9:**
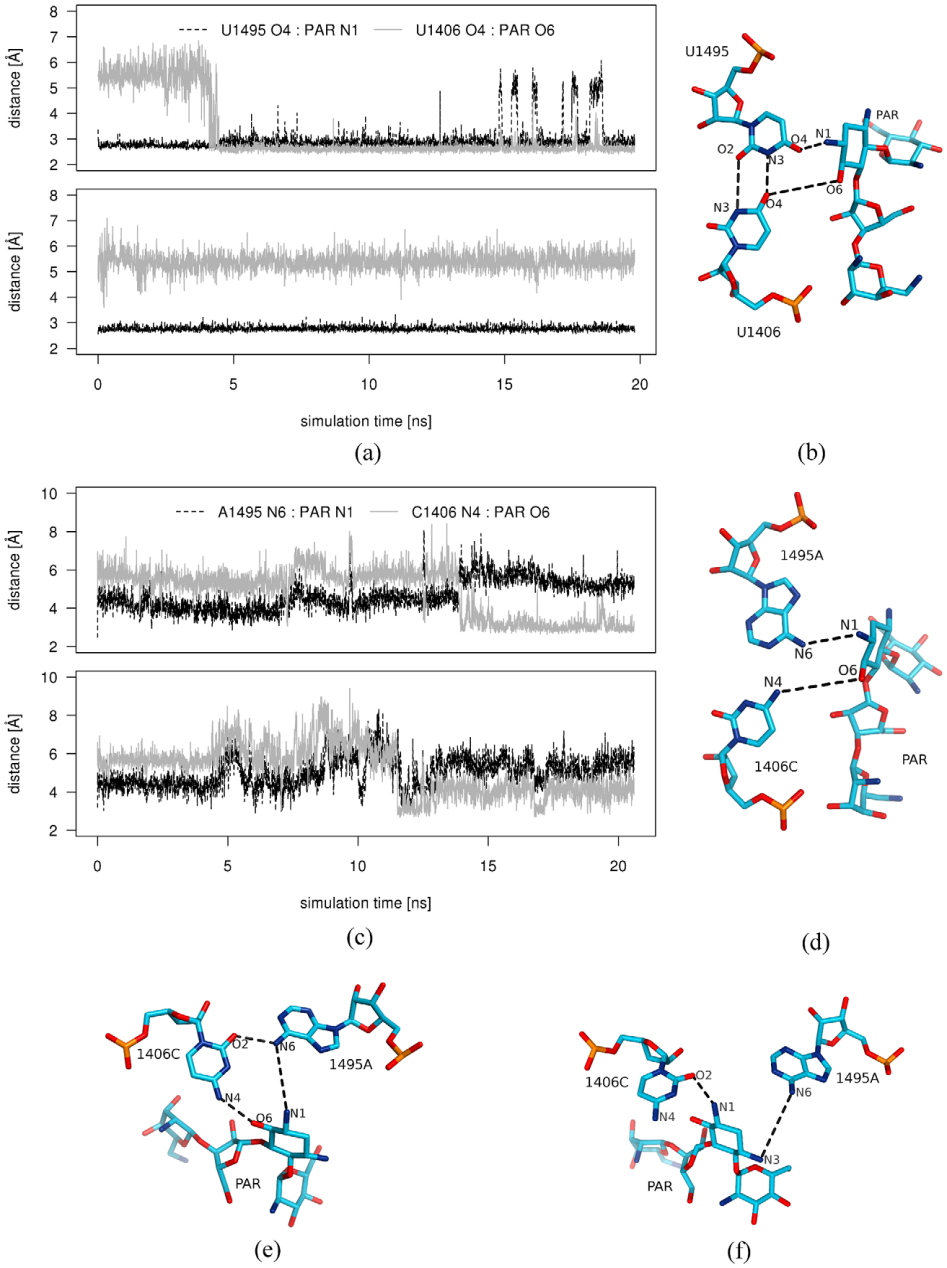
Hydrogen bonds formed by paromomycin with the RNA. Distances between the atoms of the U1406·U1495 pair and paromomycin as a function of the simulation time along with the trajectory snapshots labeling the measured distances. Data taken from simulations (a)–(b) G1491A_PAR and (c)–(f) U1406C/U1495A_PAR. Two plots depict values for the two A-sites of the crystal structure.

In addition, the position of the core of the antibiotic (rings I and II) was altered (Figure S9). Ring I moved away from the bulge and left room for A1492 and A1493. In one A-site, the PAR(N1) atom formed a hydrogen bond with 1406C(O2), which led to the disruption of the C∶A base pair (Figure 9a). In the second A-site, the C∶A pair was formed with only one hydrogen bond, and the PAR(O6) hydrogen bonded with 1406C(N4) (Figure 9b). In the non-mutated A-site [4], [29], PAR(N1) forms a tight hydrogen bond with U1495(O4) (with the distance of 2.82 Å and 2.72 Å for the two A-sites of the crystal structure [45], respectively). In addition, the bond was maintained and had a mean distance of 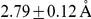 between the mentioned atoms in the MD simulation of the original complex.

Another important hydrogen bond, which is mediated by a water molecule, is formed between PAR(O6) and U1406(O4) (distances in the X-ray structure are 2.62 Å between PAR(O6) and the OW oxygen of water molecules W8 or W54; and 2.59 and 2.41 Å between W8(OW) and W54(OW), respectively, and U1406(O4); numbering of atoms as in Figure 9d). The simulations of the wild-type structure showed that towards the end of the trajectory, a direct bond was formed between RNA and paromomycin: first the distance between PAR(O6) and U1406(O4) was 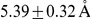, and after ca. 11 and 14 ns for each symmetrical part of the structure, respectively, it decreased to 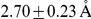. The U1406C∶U1495A mutation did not allow for the formation of the corresponding bonds (Figure 9e) and therefore could not support two very important contacts between paromomycin and RNA. These mutations almost completely prevented the proper binding of aminoglycosides in the mutated A-site, which has been shown in MIC experiments performed by Hobbie et al. [7], [8].

Paromomycin was also dynamic in the G1491A_PAR simulation. In one A-site, we observed a shift of the entire antibiotic, and ring IV rotated around the bond that formed with ring III (Figure 8c). However, this ring reorganization effect was less than in the U1406C/U1495A_PAR simulation, and the hydrogen bonds with the U1406·U1495 pair were preserved (Figure 9c). In fact, paromomycin came closer to the RNA atoms in one of the A-sites (Figures S7 and S8). The distance between U1406(O4) and PAR(O6) diminished during the simulation, like in the wild-type structure, which suggested that these atoms actually form a direct hydrogen bond.

The clustering of the conformations of the A-site in complex with paromomycin provided additional evidence that the U1406C/U1495A substitution causes the antibiotic to be less conformationally stable in the binding site and even allows for A1492 and A1493 to move into the helix (Figure S7). Moreover, we also observed differences in the range of movements of the adenines between the NON_MUT_PAR and G1491A_PAR simulations. The G1491A mutation caused the range to widen, which indicated that the bound drug may be less effective [17], [37].

### Mutations of the uridines influence the electrostatic potential of the A-site

We monitored the distribution of sodium ions and water molecules inside the binding site, since the electrostatic interactions [25] and indirect water-mediated bonding between paromomycin and RNA [4], [45] are important for the structural stability of the complex. The analysis of the distribution of ions in the MD simulations without the antibiotic can show how the mutations change the electrostatic potential of the inner side of the RNA A-site helix.

**Figure 10:**
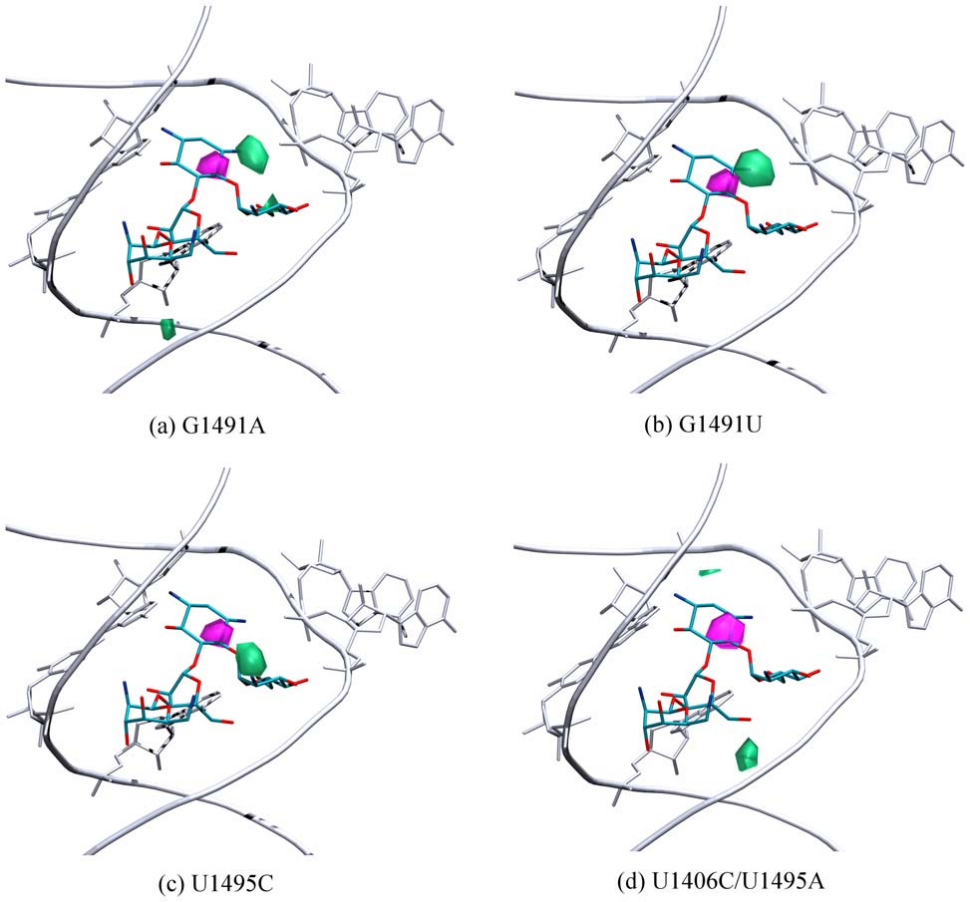
Areas of sodium ion density in the free A-site. (a)–(c) 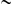
0.066 ions per 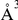, (d) 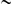
0.055 ions per 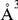; violet – MD simulations of the original structure, and green – of the mutated structures. The superimposed position of paromomycin is shown for clarity. Only the U1406·U1495 and A/G1408, A1492, A1493 bases are shown in atomic details. Hydrogen atoms were not shown for clarity of the image.

In a simulation of the wild-type prokaryotic A-site [33] the area of maximal sodium ion density (more than 0.053 ion per 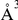) was situated in the position of the ring II of the superimposed paromomycin. The locations of high ion density areas in the G1491U and G1491A simulations were roughly similar (Figures 10a and 10b), however in comparison with the ion distribution around the wild-type structure, were shifted approximately 2 Å toward the phosphorous atom of A1493. This shift indicates that only a minor change in the electrostatic potential occurred inside the RNA bulge, which was most likely caused by A1492 and A1493 predominantly occupying the flipped-in state.

The MD simulation performed for the RNA helix with a double mutation of the U1406·U1495 pair showed larger deviations in the distribution of ions compared to the wild-type structure. Figures 10c and 10d show both of the structures in which these uracils were substituted. We noticed that the U1495C mutation introduced smaller changes than the U1406C/U1495A mutation. High sodium ion density areas in the U1495C simulation were shifted approximately 3 Å towards the major groove (Figure 10c), while in the structure with the double mutation, they were located entirely outside of the core of the binding site (Figure 10d). We noted that the ion density closest to ring II of paromomycin was not present, which provides further evidence that the U1406·U1495 pseudo-pair plays an important role in the recognition of the A-site site by aminoglycosidic antibiotics through electrostatic interactions.

### Fewer water molecules gather inside the clefts with single-point mutations

The analysis of water distribution inside the binding cleft showed that in general, the G1491U, G1491A, and U1495C were less hydrated than the wild-type structure. There were only a few dense areas observed, which can be explained by the change in the cleft shape, either by a shift of base pairing (similar to the one observed in G1491A and G1491U) or simply by A1492 and A1493 occupying the flipped-in state (which was observed in all three simulations) (Figure S10a and Table S3).

Nevertheless, we observed an area of high water density between U1406 and U1495 in both A-sites in the G1491A and G1491U simulations (in the X-ray structure this water is numbered W49; Table S3). This indicated that the U1406·U1495 pair was correctly formed, since the hydrogen bonds between these uracils are mediated by a water molecule [45]. In our previous simulations of the original prokaryotic A-site [33], these water density areas were also observed. In the U1495C simulation, where only one uracil was mutated to cytosine, there was a high water density area near U1406. This was most likely due to the fact that U1406 was often shifted towards the inside of the helix, which left space for water molecules to gather near U1406(O2) (see Figure 7d for atom numbering).

Inside the structure with the double uracil mutation, we observed more areas of high water density (Figure S10b); however, none of these were between the mutated bases, and many were located in positions where atoms of paromomycin were found after superimposing the complex structure. These data suggest that although the shape of the binding cleft is not altered in the U1406C/U1495A simulation, there are still water molecules that the antibiotic has to expel upon binding. Additionally, less positions of crystal water molecules were reconstructed in the U1406C/U1495A_PAR simulation compared to the NON_MUT_PAR simulation (6 vs. 12; Table S4). These results further confirm that paromomycin has weaker binding to the double mutated A-site.

## Discussion

In this study, we performed eight MD simulations of the model RNA fragment that contained two symmetrically positioned A-sites with various resistance-causing mutations introduced *in silico*. The simulations were carried out for systems with and without the aminoglycosidic antibiotic paromomycin. The comparative analysis of the trajectories showed differences in the physical and chemical features of the A-site that were introduced by these mutations.

### U1406·U1495 mutations affect the electrostatic potential inside the A-site

The U1406C/U1495A mutation (Figure 1a) was found to have the biggest effect on the binding site of paromomycin, which is in agreement with previous experiments that have shown changes in the minimal inhibitory concentrations (MIC) of aminoglycosides that target bacteria with different mutations in the A-site [8]. This double mutation perturbs the electrostatic potential inside the RNA helix, which in our simulations resulted in disabling the formation of proper direct and indirect contacts between paromomycin and the mutated bases.

Moreover, our simulations revealed that upon the change of base 1495 from uracil (pyrimidine) to adenine (purine), the shape of the base pair was disrupted. During the simulation, the adenine is situated more inward than the uracil in the wild-type structure, which can possibly prevent paromomycin binding by steric hindrance. This apparent conformational change of the mutated base pair did not seem to affect the other base pairs' stability, and throughout the simulation, the A-site model retained its overall structure, which is in agreement with a previous study [46].

In all of the MD simulations of the complexes with the antibiotic, with the exception of U1406C/U1495A_PAR, paromomycin was firmly bound to RNA, and the complex was less conformationally dynamic than RNA alone. In contrast, the U1406C/U1495A_PAR simulation showed that the drug changed its conformation and slid out from the binding cleft, which indicated that the hydrogen bonds formed with the mutated structure were not stable. When comparing the simulations of structures with mutations of the U·U pair, we noticed that the U1495C substitution has a smaller overall effect than the U1495A substitution, which is in agreement with the experimental studies on affinities of paromomycin for ribosomal 30S subunits that possess different mutations in the A-site [6].

Our studies, together with other works where more uracil mutations have been tested [6], [8] suggest that the negative electrostatic potential created by base 1495 may be more important for proper recognition of aminoglycosides than the geometry of this base pair. The double mutation in this study completely disrupted both features of the base pair, while the U1495C preserved the shape and one of the negatively charged moieties. The U1406C/U1495G mutation previously examined by Hobbie *et al.* maintained only the negative charge distribution on the 1495 base. The U1406C/U1495G substitution had almost no effect on the MIC value, which was elevated for the other two mutations (U1406C/U1495A and U1495C).

### The A-site bulge changes its shape due to 1491 mutations

Mutation of the G1491 base also induced a significant effect on the A-site. Previous studies have shown that mutation of G1491 to U and A conferred high levels of resistance to paromomycin [8], [11]. In the G1491U and G1491A simulations, we observed a shift in the base pairing, including the mutated base. This shift changed the internal dynamics of the binding site and enabled A1492 and A1493 to occupy the flipped-in state for a longer period of time, which could lead to steric clashes with paromomycin and preclude its accommodation in the A-site. Steric changes may influence the ability of aminoglycosides to bind, and may have an even larger effect than changes in the electrostatic potential [47]. Hobbie *et al.* has suggested that the intra-helical side of adenine is less nucleophilic than that of guanine, and therefore the G1491A substitution diminishes the strength of the hydrogen bonds formed with the drug [7]. Our results show that this mutation significantly changes the shape of the cleft to a point where paromomycin may have difficulty fitting into the binding site.

A previous study of the *eukaryotic* yeast A-site showed that the A1491G substitution only caused a slight decrease in translation error frequency [39]; however, it was shown to increase 10-fold in the presence of the antibiotic. Therefore, the reverse mutation in bacteria can reduce the effect of the bound aminoglycoside at the expense of a slight increase in the translation error rate in the absence of the drug. Our G1491A_PAR simulation showed that A1492 and A1493 acquire conformations close to the flipped-in state, which corresponds to the decreased effectiveness of the antibiotic [17], [37]. Moreover, in the simulation without the drug (G1491A), these adenines stayed in the flipped-in state for a longer period of time than in the wild-type structure, indicating a possible increase in translation errors, which could occur by rejecting too many tRNA molecules.

The MD simulations presented in this study also suggested a cause for the increased probability of a stop codon read-through due to the G1491A mutation that was previously reported [36]. In comparison to the NON_MUT simulation, we noticed that A1492 and A1493 were almost never apart in the G1491A simulation. However, in order to correctly recognize the termination factor, A1493 must stay inside the A-site RNA helix and A1492 must be flipped-out to form the necessary contacts [20], [21]. Therefore, changes in the movement of the adenines introduced by the G1491A substitution reduces the probability that the termination factor will be accepted.

It has been hypothesized that the mutation G1491U is more evolutionary profitable than the G1491A substitution [48]. In this paper, we showed that the simulation G1491A brings more changes to the A-site model and that the flipped-in conformations of the adenines A1492 and A1493 are much more stable in this simulation than in the G1491U simulation and the wild-type structure. This may explain the worse “fitness” of bacteria possessing a G1491A substitution.

Moreover, we observed that the complexes of paromomycin with either of the G1491-mutated structures are quite stable, suggesting that in this case, the resistance comes from the smaller percentage of binding-enabled conformations of the A-site in the dynamic ensemble. It has been previously shown with combined experimental and theoretical approaches [38] that in case of aminoglycosides and the ribosome, binding is achieved through so-called stochastic gating or conformational selection, and not an induced fit mechanism. Therefore, the drug simply has a much lower probability of finding a G1491A mutated A-site in a favorable conformation.

### Proposed modifications of paromomycin

We propose that an alteration of the substituent at the 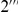 position of paromomycin ring IV (Figure 1b) may improve the binding in the A-site, even with mutations of the U1406·U1495 bases. Specifically, substitution of the 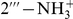 group with the 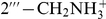 would allow the ring IV to interact with phosphate groups of both G1405 and U1490 or G1491. In the G1491A_PAR and G1491U_PAR simulations a bond was formed between 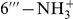 and O2P of the mutated base 1491, or even with U1490(O2P). It existed either in place of or along with the hydrogen bond between 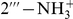 of paromomycin and G1405(O2P). This was most likely due to the change in the shape of the binding cleft in these simulations, since we did not observe the former interaction in the other simulations, especially in the NON_MUT simulation. However, with the proposed extension of the 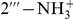 paromomycin, ring IV may always be hydrogen bonded to both sides of the major groove, which would anchor the drug even more.

Similarly, the 6-OH group of ring II, which forms a water-bridged hydrogen bond with U1406(O4) in the unmodified A-site [29], [45], could be substituted for 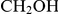 group. Thus, it may form a direct hydrogen bond with the unmodified base U1406. Moreover, if the amino group at 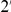 position (ring I) was switched with the OH substituent at 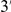 position, the bonds formed with the phosphate group of A1492 could be tighter, which would therefore anchor the neamine part of the drug more (this interaction was weak in the G1491A and the U1406C/U1495A simulations).

Important hydrogen bonds are also formed with the 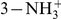 group (ring II), but they are not stable in simulations of the structures with mutated U1406·U1495 bases. We have noticed that the distance between the nitrogen N3 of paromomycin and phosphorous atom of either G1494 or A1493 is quite big after the equilibration (an increase from 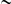
3.9 Å up to 4.9 Å). Therefore, an extension at this position (i.e., 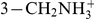 instead of a simple amino group) could improve binding, which may also diminish the effect of the double mutation U1406C/U1495A.

## Methods

### Starting structures and system preparation

We used a 44-nucleotide RNA model containing two symmetrically positioned A-sites that were complexed with paromomycin as the starting structure (Figure 1a depicts half of the sequence of the model, Figure 1b shows the structure of paromomycin; PDB code of the whole structure: 1J7T [45], 2.5 Å-resolution). This rRNA region forms a helix with a bulge created by the following three adenines: A1408, A1492, and A1493. The chosen model proved to be a good representative of the original binding site, which is a solvent-exposed region in the small ribosomal subunit [33], [49], [50]. Since the model is deprived of the influence of all the surrounding ribosomal RNA and proteins that exist in the complete ribosome assembly, it could be questioned whether the behavior of the nucleic bases, particularly of the two adenines A1492 and A1493, can be reliably represented. Therefore, we have compared the solvent accessible surface area (SASA) of these adenines in different X-ray structures of the ribosome with the values from the simulation of the model (Figure S11; values were calculated with VMD software [51]). The range of the values obtained from the simulation of the wild-type A-site model were within the range calculated for the experimental static structures. In this study we also investigate the geometry and dynamics of the U1406·U1495 base pair. In the whole 70S ribosome it is involved in some tertiary contacts (A1919 of 23S rRNA and G1517 of 16S rRNA; see e.g., structures 3I8F and 3I8G [52]), which we are not able to mimic in our model. Nevertheless, these bases form a stable pair in the wild-type structure [33] and we did not observe bulged-out conformations of either U1406 or U1495. These results provided additional reassurance that the model in our simulations can reliably reproduce the shape and internal dynamics of the A-site inside the 30S ribosomal subunit.

Mutations were chosen based on previous experimental studies [7], [10], [11] and were introduced using the Sybyl (Tripos) software. We believe that a well-established protocol that includes minimization followed by heating and equilibration of the whole system (described below) yields a valid starting structure for the further collection of the production phase data. We also performed simulations of the RNA models with and without the drug, in order to have a reference when seeking changes in the features of the binding site that resulted from the presence of the bound antibiotic. All of the types of MD simulations together with their abbreviations used in the text are listed in Table 1.

The system was neutralized by adding sodium ions 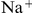 around the molecule with the use of LEaP from the Amber9 package [53]. In this step 44 and 34 ions were added to the structures without and with paromomycin, respectively. The neutralized molecules were then submerged into boxes of TIP3P [54] water molecules, again with the use of the LEaP program. The dimensions of each system were 92×69×69 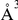. Finally, random water molecules were substituted with 39 sodium (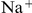, radius: 1.5 Å, mass: 22.99 a.u.) and 39 chlorine (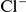, radius: 1.5 Å, mass: 35.45 a.u.) ions, in order to obtain an ionic strength of approximately 150 mM. Sodium ions were chosen because they were better represented in the force field that was used than potassium ions, for example (see ref. [29], [55]). The Amber ff99 [56] force field was selected for the RNA. A newer version of this force field is available, called parmbsc0 [57], however we did not use it since we wanted to compare the results with our previous simulations that utilized the ff99. Moreover, recent studies showed that there is little difference between these types of parametrization in relation to RNA simulations [58], [59]. Very recently, some improvements of RNA force field parameters were proposed [60]. Banaš *et al.* have shown that even in the parmbsc0 force field, the 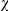 angle (i.e., the dihedral angle of the linkage between the ribose and the nucleic base) may adopt some non-standard values, leading to a so-called “ladder-like” structure formation instead of a normal A-RNA helix. We are aware that the parametrization of the RNA force field is far from perfect; however, on short timescales (similar to our 20-ns trajectories) and for simple tertiary structures (i.e., helical RNA) it has been proven through many simulations that the experimental fluctuations and the overall structure is maintained [15], [58]. In addition, the 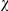 angle in our simulations behaved well for all the RNA sequences and we did not observe high-*anti* conformations (see Figure S12). Moreover, our trajectories were not as long as those tested in ref. [60], and therefore we believe that the conformational changes reported here do not result from an improper parametrization of RNA.

The parameters for paromomycin were created with the use of antechamber program from Amber suite, using GAFF [61] force field and AM1BCC charges. Since this is an automatic approach, with no guarantee for yielding correct force field parameters, we further tested the force field parameters. We performed two 10-ns long MD simulations of paromomycin in water (with different initial velocities) and compared some conformational features to the existing NMR data [44] (see Figures S13 and S14). The work of Asensio *et al.* analyzed NMR spectra of an aminoglycoside neomycin, which differs from paromomycin in only one chemical group (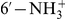 in place of 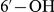). This did not seem to influence the flexibility of the drug, as our simulations showed a very good correlation with the NMR-derived data. The complete parameters are given in Supplementary Dataset S1.

### Simulation protocol

The computational protocol was essentially the same as previously described [33]. Briefly, the energy minimization was carried out with the sander program of the Amber9 package. Afterwards, the simulations were performed with NAMD [62] under constant pressure (using the Langevin piston method [63]) and temperature (controlled by Langevin thermostat [64]) and with periodic boundary conditions. Electrostatic interactions were calculated using the Ewald Summation method (PME [65]). The SHAKE [66] algorithm was used which allowed for a 2 fs simulation time step. Thermalization from 30–310 K was performed with constraints applied to all heavy atoms of the RNA and, if applicable, paromomycin. The constraint coefficient (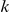) was equal to 50 
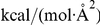
 for the first 85 ps of simulation and then 25 
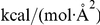
 for another 35 ps. The constraints were then gradually weakened during first 300 ps of the equilibration stage. For the remaining 600 ps, the constraints were applied only to heavy atoms of the terminal nucleotides C1402 (Figure 1a; 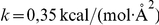, to 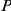 atoms of C1498 (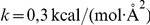, and to 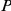 atoms of G1403 (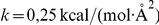. These values were adjusted so as to obtain the fluctuations of the termini that corresponded to the crystallographic temperature factors. The MD production stage was performed under the same conditions as the second part of equilibration and lasted 20 ns.

In general, the MD simulations performed under constant temperature only sample configurations that are close to the energetical minimum of the given biomolecule, and do not enable crossing larger energetical barriers. Therefore, the trajectory can be quite limited. In our study, each of the simulations was quite short (20 ns), but the system includes two symmetrical binding sites, which enlarge the conformational sampling space. In addition, the analysis was mainly comparative between the structures with different mutations. We did not specifically gather statistics on nucleobase flipping.

### Data analysis

We used standard measures to test the conformational stability of molecules, including the root mean square deviation (RMSD) of atomic positions from the initial structure and the root mean square fluctuations (RMSF) of each residue. These were calculated with the g_rmsf and g_rms programs from the GROMACS package [67]–[69] for all non-hydrogen atoms.

3DNA software [70] was used to monitor hydrogen bonds between the paired bases and the opening angle of the base pairs. The detailed description of the method can be found in the software manual, which is available at http://rutchem.rutgers.edu/~xiangjun/3DNA.

For the description of A1492 and A1493 flipping, we define the pseudo-dihedral angle 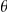 as the torsion angle between the lines connecting the four atoms G1494(N1), G1494(P), A1492/3(P), and A1492/3(N1) (Figure S3). Base 1494 was stably positioned in all simulations and formed a pair with an opposite C1409. Therefore, it served as a good reference for the flipped-in conformation. For this study, we defined the flipped-in state of A1492 and A1493 when 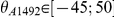 and 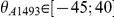, respectively. All other values of 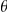 point to the base being outside of the RNA helix. A similar measure has been employed in other studies to measure the conformational variation of bases in RNA [16], [71] and DNA [72], [73].

The clustering of conformations obtained from MD simulations was performed by the ptraj program of AmberTools (version 1.3, available at http://ambermd.org). Each trajectory was aligned with the first frame in order to eliminate translations and rotations of the structure. The terminal residues C1402 were not considered during clustering because of the applied constraints. The average linkage method was chosen with a maximum of five clusters, and the clustering of the structures was performed according to the RMS distance measure for all heavy atoms. Other maximal numbers of clusters were tested, however the former settings gave optimal results.

The calculation of sodium ions and water distribution inside the binding cleft was performed with the use of MolDyAna software (http://moldyana.icm.edu.pl/moldyana; see also Methods section in [33]). VMD [51] and The PyMol Molecular Graphics System (Schrödinger, LLC., http://www.pymol.org) were used to visualize trajectories and R environment (http://www.R-project.org) in order to produce plots.

## Supporting Information

Dataset S1 **Topology file made with the use of LEaP program of Amber suite, consisting all the parameters of paromomycin.**
(PDF)

Figure S1 **Distances between the atoms of paromomycin and RNA bases [Å] **in the NON_MUT_PAR simulation and in the X-ray structure (PDB code: 1J7T) [Vicens, Q.; Westhof, E. *Structure* **. 2001, **9, 647–58].
(PDF)

Figure S2 **Average RMSD [Ångstrom]** with their standard deviations; two graphs depict RMSD for two A-sites.
(PDF)

Figure S3
**Pseudo-dihedral angles describing the variation in the conformations of A1492 and A1493.** The angle 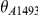 is calculated as a torsion angle between the four atoms: G1494(N1) – G1494(P) – A1493(P) – A1493(N1), as depicted by black sticks (analogously for 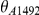: G1494(N1) – G1494(P) – A1492(P) – A1492(N1)). The exemplary snapshot is taken from the NON_MUT simulation, with the values of the pseudo-dihedral angles (in degrees) shown in brackets.(PDF)

Figure S4 **Conformations of bases forming the A-site in the most populated clusters in different MD simulations.** The distances (in Å) between chosen atoms are shown as black dotted lines; two snapshots per simulation correspond to two A-sites in the crystal structure; for base numbering see the inset in the Figure S5 and Figure 1a.
(PDF)

Figure S5 **Distances between chosen atoms inside the A-site as a function of the simulation time.** Four sets of graphs correspond to four distances (1, 2, 3, 4) depicted as black dotted lines on a stick model of the A-site fragment; for base numbering see Figure 1a. Grey and black lines are for two simulated A-sites.
(PDF)

Figure S6 **Representative structures of the clusters for different MD simulations of the free A-site RNA, superposed with regard to phosphorous atoms.** The structures from each cluster are colored differently: 1 – green, 2 – blue, 3 – red, 4 – yellow, 5 – cyan (see Table S1 for the cluster sizes). Adenines A1492, A1493 and A1408 are shown in atomic detail.
(PDF)

Figure S7 **Representative structures of the clusters for different MD simulations of the A-site in the complex with paromomycin, superposed with regard to phosphorous atoms.** Structures from each cluster are colored differently: 1 – green, 2 – blue, 3 – red, 4 – yellow, 5 – cyan (see Table S1 for the cluster sizes). Paromomycin and A1492, A1493 and A1408 are shown in atomic detail.
(PDF)

Figure S8 **Distances between the centers of mass of adenine phosphorous atoms, A1492(P) and A1493(P), and paromomycin ring III (** ***left*** **) or ring IV (** ***right*** **).** The frequency distributions of the distances are shown next to each graph. Black and grey lines correspond to the two A-sites of the crystal structure.
(PDF)

Figure S9 **Distances between the centers of mass of adenine phosphorus atoms, A1492(P) and A1493(P), and paromomycin ring I (** ***left*** **) or ring II (** ***right*** **).** The frequency distributions of the distances are shown next to each graph. Black and grey lines correspond to the two A-sites of the crystal structure.
(PDF)

Figure S10
**Areas of high water density **(light blue, 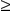 **0.23 water oxygens per** 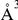 **) located in one part of the simulated structure, superposed on the crystal structure of the complex with paromomycin (PDB entry 1J7T). Only the U1406·U1495 and A/G1408, A1492, A1493 bases are shown in atomic details; spheres show the positions of the crystal water oxygen atoms, the ones which were identified in the simulation are marked in orange (see also Table S3). Hydrogen atoms were not shown for clarity of the image.**
(PDF)

Figure S11
**Solvent accessible surface area (SASA) [** 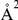 **] of the two adenines A1492 and A1493, **calculated for the ribosome structures available in PDB in higher resolution (i.e., structures containing only phosphorous atoms were discarded). The values are compared with an average and standard deviation calculated for the NON_MUT simulation (for both symmetrical parts of the model structure, without taking into account the hydrogen atoms, since X-ray structures do not contain these).(PDF)

Figure S12
**Average glycosidic torsion angle** 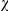 **, **calculated for all the residues in each simulation per each frame. The scale on y axes spans the possible range of 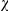 (−180, +180); compare with Figure 6 in [Banáš, P. *et al.*, Journal of Chemical Theory and Computation. **2010**, 6, 3836–3849].(PDF)

Figure S13
**Dihedral angles of linkages between the rings of paromomycin.** The angles 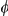 and 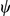 are defined as in [Asensio, J. L. *et al.*, Chemistry. **2002**, 8, 5228–40], an NMR study of neomycin, which differs from paromomycin only with one chemical group, having OH instead of 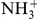. The values were collected during two independent 10-ns production phases of MD of paromomycin in water (the two runs started from the same minimized structure but with different initial velocities).(PDF)

Figure S14
**Distances between chosen hydrogen atoms of paromomycin, **compared with values from an NMR study of neomycin, which differs from paromomycin only with one chemical group, having OH instead of NH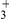 . The experimental values are taken from Table 1 in [Asensio, J. L. *et al.*, Chemistry. **2002**, 8, 5228–40] and are as follows: 1 – H1Glc–H4Strp, 2 – H1Glc–H5Strp, 3 – H1Glc–H3Strp, 4 – H1Glc–H5Rib,5 – H1Glc–H2Rib, 6 – H1Glc–H3Rib, 7 – H1Rib–H5Strp, 8 – H1Rib–H4Strp, 9 – H1Rib–H6Strp,10 – H2Rib–H6Strp, 11 – H1Rib–H4Rib, 12 – H1Ido–H3Rib, 13 – H1Ido–H2Rib, 14 – H1Ido–H4Rib. The asterisks (*) mark the distances that were described as larger than in the experimental work.
(PDF)

Table S1 **Distribution of the MD conformations into clusters.** Occupancy of each of the clusters derived from different MD simulations; the cluster with the highest population (occupancy) is underlined.
(PDF)

Table S2
**Base pairing.** Percentage of simulation time when base pairs were formed (i.e., at least one hydrogen bond was present). Two values are shown for each simulation corresponding to two A-sites. 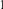 data from our previous study (Romanowska J., Setny P., Trylska J., *J. Phys. Chem. B* **2008**); 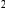 this base is U in G1491A, G1491U, NON_MUT and U1495C; and C in U1406C/U1495A simulation; 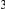 this base is U in G1491A, G1491U and NON_MUT; C in U1495C; and A in U1406C/U1495A simulation; 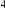 this base is A in G1491A, and U in G1491U.(PDF)

Table S3
**Reproduction of the crystal water molecules in simulations of the free A-site.** Selected water molecules of the 1J7T crystal structure and the corresponding water density areas in MD simulations without the antibiotic. “
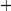
” denotes water density areas higher than 0.22 water molecules per 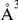 observed in the position of the corresponding crystallographic water molecule; “
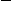
” denotes lack of high water density in this position. Brackets denote analogous water molecules located in the other symmetric part of the RNA fragment.(PDF)

Table S4
**Reproduction of the crystal water molecules in simulations of the complexed A-site.** Selected water molecules of the 1J7T crystal structure and the corresponding water density areas in the MD simulations with paromomycin. “
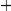
” denotes water density areas higher than 0.22 water molecules per 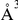 observed in the position of the corresponding crystallographic water molecule; “
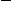
” denotes lack of high water density in this position. Brackets denote analogous water molecules located in the symmetric part of the RNA fragment.(PDF)
