# Supplementary material for: Understanding the Origins of Bacterial Resistance to Aminoglycosides through Molecular Dynamics Mutational Study of the Ribosomal A-Site
Source: PLoS Comput Biol. 2011 Jul 21;7(7):e1002099. doi: 10.1371/journal.pcbi.1002099 (PMC3140962; doi:10.1371/journal.pcbi.1002099)
Supplement: Dataset S1 — Topology file made with the use of LEaP program of Amber suite, consisting all the parameters of paromomycin. (PDF) [file pcbi.1002099.s001.pdf]

Below is the topology file made with the use of LEaP program of Amber suite, consisting all the parameters of paromomycin.

```
%VERSION  VERSION_STAMP = V0001.000  DATE = 03/07/11  12:02:16
%FLAG TITLE
%FORMAT(20a4)

%FLAG POINTERS
%FORMAT(10I8)
      92      10      50      45     115      67     229     164       0       0
     552       1      45      67     164      10      20      16      10       0
       0       0       0       0       0       0       0       0      92       0
       0
%FLAG ATOM_NAME
%FORMAT(20a4)
O42 HO42C42 C32 C22 H22 N22 HN12HN22HN32H32 O32 HO32H42 C52 H52 C62 H612H622O62
HO62O52 C12 H12 O12LC41 H411C31 H311N31 HM11HM21HM31C21 H211H221C11 H111N11 HN11
HN21HN31C61 H611O61 HO61C51 H511O13LC13 H123C23 H23 O23 HO23O43 C43 H43 C53 O53
HO53H513H523C33 H33 O34LC14 H14 O54 C54 H54 C64 H614H624N64 HM14HM24HM34C44 H44
O44 HO44C34 H34 O34 HO34C24 H24 N24 HN14HN24HN34
%FLAG CHARGE
%FORMAT(5E16.8)
 -9.65800122E+00  7.83577122E+00  1.75353193E+00  1.75079858E+00  1.55290441E+00
  2.79985640E+00 -1.49215126E+01  8.67745926E+00  8.30426656E+00  9.14668349E+00
  1.29651665E+00 -1.10791584E+01  8.91781140E+00  2.52889079E+00  1.53577544E+00
  9.92750904E-01  2.42793925E+00  1.70396727E+00  2.09319560E+00 -1.27206232E+01
  8.68183261E+00 -8.16176817E+00  5.96598102E+00  2.08135111E+00 -8.63226796E+00
  2.56241983E+00  1.88582583E+00  2.09465338E+00  2.46766387E+00 -1.52121583E+01
  8.80683759E+00  8.64484134E+00  9.24180389E+00 -2.53308192E+00  2.07825331E+00
  1.89165696E+00  2.34703224E+00  2.55385535E+00 -1.52105183E+01  8.93530481E+00
  9.11424779E+00  8.61040120E+00  1.80783438E+00  1.62397138E+00 -1.10802517E+01
  8.39647139E+00  2.10977789E+00  1.89985700E+00 -7.87002915E+00  5.89746517E+00
  2.06221769E+00  1.42534831E+00  1.79653656E+00 -1.08679619E+01  7.99084300E+00
 -8.35401344E+00  1.96946618E+00  1.94249718E+00  2.28471197E+00 -1.17838147E+01
  8.33816003E+00  1.38070367E+00  1.37359697E+00  1.93356825E+00  1.76482976E+00
 -7.61746807E+00  6.15039070E+00  2.02868866E+00 -8.34472006E+00  2.13182688E+00
  2.34830780E+00  1.93739494E+00  2.45490826E+00  2.70892712E+00 -1.51729803E+01
  8.70133047E+00  8.40704033E+00  8.98250056E+00  1.30544557E+00  2.27013413E+00
 -1.20857583E+01  8.64156133E+00  2.08554224E+00  1.89220363E+00 -1.08905576E+01
  8.71171718E+00  1.43883281E+00  2.71530492E+00 -1.50940778E+01  8.70661494E+00
  8.73905063E+00  8.76437963E+00

%FLAG MASS
%FORMAT(5E16.8)
  1.60000000E+01  1.00800000E+00  1.20100000E+01  1.20100000E+01  1.20100000E+01
  1.00800000E+00  1.40100000E+01  1.00800000E+00  1.00800000E+00  1.00800000E+00
  1.00800000E+00  1.60000000E+01  1.00800000E+00  1.00800000E+00  1.20100000E+01
  1.00800000E+00  1.20100000E+01  1.00800000E+00  1.00800000E+00  1.60000000E+01
  1.00800000E+00  1.60000000E+01  1.20100000E+01  1.00800000E+00  1.60000000E+01
  1.20100000E+01  1.00800000E+00  1.20100000E+01  1.00800000E+00  1.40100000E+01
  1.00800000E+00  1.00800000E+00  1.00800000E+00  1.20100000E+01  1.00800000E+00
  1.00800000E+00  1.20100000E+01  1.00800000E+00  1.40100000E+01  1.00800000E+00
  1.00800000E+00  1.00800000E+00  1.20100000E+01  1.00800000E+00  1.60000000E+01
  1.00800000E+00  1.20100000E+01  1.00800000E+00  1.60000000E+01  1.20100000E+01
  1.00800000E+00  1.20100000E+01  1.00800000E+00  1.60000000E+01  1.00800000E+00
  1.60000000E+01  1.20100000E+01  1.00800000E+00  1.20100000E+01  1.60000000E+01
  1.00800000E+00  1.00800000E+00  1.00800000E+00  1.20100000E+01  1.00800000E+00
  1.60000000E+01  1.20100000E+01  1.00800000E+00  1.60000000E+01  1.20100000E+01
```

```
  1.00800000E+00  1.20100000E+01  1.00800000E+00  1.00800000E+00  1.40100000E+01
  1.00800000E+00  1.00800000E+00  1.00800000E+00  1.20100000E+01  1.00800000E+00
  1.60000000E+01  1.00800000E+00  1.20100000E+01  1.00800000E+00  1.60000000E+01
  1.00800000E+00  1.20100000E+01  1.00800000E+00  1.40100000E+01  1.00800000E+00
  1.00800000E+00  1.00800000E+00
%FLAG ATOM_TYPE_INDEX
%FORMAT(10I8)
       1       2       3       3       3       4       5       6       6       6
       7       1       2       7       3       7       3       7       7       1
       2       8       3       9       8       3       7       3       4       5
       6       6       6       3      10      10       3       4       5       6
       6       6       3       7       1       2       3       7       8       3
       9       3       7       1       2       8       3       7       3       1
       2       7       7       3       7       8       3       9       8       3
       7       3       4       4       5       6       6       6       3       7
       1       2       3       7       1       2       3       4       5       6
       6       6
%FLAG NUMBER_EXCLUDED_ATOMS
%FORMAT(10I8)
      11       4      16      17      15      10       9       3       2       1
       5       4       1       4      10       6       6       4       3       2
       1       4       6       2      10      18       8      15       9       8
       3       2       1      12       5       4      12       8       7       3
       2       1       7       5       4       1       6       2       8      12
       6      11       7       6       1       9      10       7       7       4
       2       2       1       6       2       8      15       6      13      16
       8      10       6       5       4       2       1       1      10       6
       5       1       9       5       4       1       5       4       3       2
       1       1
%FLAG NONBONDED_PARM_INDEX
%FORMAT(10I8)
       1       2       4       7      11      16      22      29      37      46
       2       3       5       8      12      17      23      30      38      47
       4       5       6       9      13      18      24      31      39      48
       7       8       9      10      14      19      25      32      40      49
      11      12      13      14      15      20      26      33      41      50
      16      17      18      19      20      21      27      34      42      51
      22      23      24      25      26      27      28      35      43      52
      29      30      31      32      33      34      35      36      44      53
      37      38      39      40      41      42      43      44      45      54
      46      47      48      49      50      51      52      53      54      55
%FLAG RESIDUE_LABEL
%FORMAT(20a4)
PAR
%FLAG RESIDUE_POINTER
%FORMAT(10I8)
       1
%FLAG BOND_FORCE_CONSTANT
%FORMAT(5E16.8)
  3.69000000E+02  3.38700000E+02  2.93600000E+02  3.69600000E+02  3.35900000E+02
  3.14100000E+02  3.03100000E+02  3.01500000E+02  3.26400000E+02  3.37300000E+02
%FLAG BOND_EQUIL_VALUE
%FORMAT(5E16.8)
  1.03300000E+00  1.09100000E+00  1.49900000E+00  9.74000000E-01  1.09300000E+00
  1.42600000E+00  1.53500000E+00  1.43900000E+00  1.10000000E+00  1.09200000E+00
%FLAG ANGLE_FORCE_CONSTANT
%FORMAT(5E16.8)
  4.05200000E+01  4.90200000E+01  4.61900000E+01  6.77200000E+01  5.09700000E+01
```

```
  4.63600000E+01  4.70900000E+01  4.60200000E+01  6.44500000E+01  6.32100000E+01
  3.90400000E+01  6.77800000E+01  5.08400000E+01  5.08400000E+01  4.60200000E+01
  6.23900000E+01  7.17200000E+01  3.91800000E+01  4.63700000E+01  3.94300000E+01
%FLAG ANGLE_EQUIL_VALUE
%FORMAT(5E16.8)
  1.88687626E+00  1.88338560E+00  1.92178286E+00  1.90991462E+00  1.91776860E+00
  1.92108473E+00  1.88774893E+00  1.95023174E+00  1.99526126E+00  1.93085858E+00
  1.93277844E+00  1.89228679E+00  1.89926811E+00  1.89507931E+00  1.94761375E+00
  1.96262359E+00  1.92405179E+00  1.91200902E+00  1.92073567E+00  1.89106506E+00
%FLAG DIHEDRAL_FORCE_CONSTANT
%FORMAT(5E16.8)
  1.55555556E-01  2.50000000E-01  1.60000000E-01  1.66666667E-01  0.00000000E+00
  1.17500000E+00  1.44000000E-01  2.00000000E-01  2.50000000E-01  1.80000000E-01
  1.00000000E-01  3.83000000E-01  3.83333333E-01  1.35000000E+00  8.50000000E-01
  1.00000000E-01
%FLAG DIHEDRAL_PERIODICITY
%FORMAT(5E16.8)
  3.00000000E+00  1.00000000E+00  3.00000000E+00  3.00000000E+00  3.00000000E+00
  2.00000000E+00  3.00000000E+00  1.00000000E+00  2.00000000E+00  3.00000000E+00
  2.00000000E+00  3.00000000E+00  3.00000000E+00  1.00000000E+00  2.00000000E+00
  3.00000000E+00
%FLAG DIHEDRAL_PHASE
%FORMAT(5E16.8)
  0.00000000E+00  0.00000000E+00  0.00000000E+00  0.00000000E+00  0.00000000E+00
  0.00000000E+00  0.00000000E+00  3.14159400E+00  3.14159400E+00  0.00000000E+00
  3.14159400E+00  0.00000000E+00  0.00000000E+00  3.14159400E+00  3.14159400E+00
  0.00000000E+00
%FLAG SOLTY
%FORMAT(5E16.8)
  0.00000000E+00  0.00000000E+00  0.00000000E+00  0.00000000E+00  0.00000000E+00
  0.00000000E+00  0.00000000E+00  0.00000000E+00  0.00000000E+00  0.00000000E+00
%FLAG LENNARD_JONES_ACOEF
%FORMAT(5E16.8)
  5.81803229E+05  0.00000000E+00  0.00000000E+00  7.91544157E+05  0.00000000E+00
  1.04308023E+06  1.45985502E+04  0.00000000E+00  2.27401052E+04  2.01823541E+02
  7.44975864E+05  0.00000000E+00  9.95480466E+05  2.01791425E+04  9.44293233E+05
  1.40467023E+03  0.00000000E+00  2.56678134E+03  9.14716912E+00  2.12601181E+03
  1.39982777E-01  4.66922514E+04  0.00000000E+00  6.78771368E+04  8.79040886E+02
  6.20665997E+04  5.94667300E+01  3.25969625E+03  4.58874091E+05  0.00000000E+00
  6.28541240E+05  1.11851919E+04  5.89818288E+05  1.03954408E+03  3.63097246E+04
  3.61397723E+05  3.15360051E+04  0.00000000E+00  4.68930885E+04  5.37190941E+02
  4.24594555E+04  3.20009193E+01  2.09814978E+03  2.44050579E+04  1.32801250E+03
  6.82786631E+04  0.00000000E+00  9.71708117E+04  1.41077189E+03  8.96776989E+04
  1.07193646E+02  4.98586848E+03  5.33379252E+04  3.25969625E+03  7.51607703E+03
%FLAG LENNARD_JONES_BCOEF
%FORMAT(5E16.8)
  6.99746810E+02  0.00000000E+00  0.00000000E+00  6.93079947E+02  0.00000000E+00
  6.75612247E+02  5.79323581E+01  0.00000000E+00  6.13981767E+01  3.56012899E+00
  7.50714425E+02  0.00000000E+00  7.36907417E+02  6.45756063E+01  8.01323529E+02
  1.79702257E+01  0.00000000E+00  2.06278363E+01  7.57919667E-01  2.09604198E+01
  9.37598976E-02  1.03606917E+02  0.00000000E+00  1.06076943E+02  7.42992380E+00
  1.13252061E+02  1.93248820E+00  1.43076527E+01  5.89183300E+02  0.00000000E+00
  5.85549272E+02  4.80771660E+01  6.33305958E+02  1.46567808E+01  8.66220817E+01
  4.95732238E+02  8.51470647E+01  0.00000000E+00  8.81685417E+01  5.80823477E+00
  9.36708259E+01  1.41762397E+00  1.14788417E+01  7.10161395E+01  9.13231543E+00
  1.25287818E+02  0.00000000E+00  1.26919150E+02  9.41257003E+00  1.36131731E+02
  2.59456373E+00  1.76949863E+01  1.04986921E+02  1.43076527E+01  2.17257828E+01
%FLAG BONDS_INC_HYDROGEN
```

```
%FORMAT(10I8)
     264     267       1     264     270       1     264     273       1     258
     261       2     252     255       4     246     249       5     240     243
       4     234     237       5     222     225       1     222     228       1
     222     231       1     213     216       2     213     219       2     207
     210       5     198     201       9     189     192       5     177     180
       4     174     183       5     174     186       5     168     171       5
     159     162       4     153     156       5     147     150       9     138
     141       5     132     135       4     126     129       5     114     117
       1     114     120       1     114     123       1     108     111       2
      99     102      10      99     105      10      87      90       1      87
      93       1      87      96       1      81      84       2      75      78
       5      66      69       9      57      60       4      48      51       5
      48      54       5      42      45       5      33      36       4      18
      21       1      18      24       1      18      27       1      12      15
       2       9      30       5       6      39       5       0       3       4
%FLAG BONDS_WITHOUT_HYDROGEN
%FORMAT(10I8)
     258     264       3     246     252       6     246     258       7     234
     240       6     234     246       7     213     222       3     207     213
       7     207     234       7     204     207       8     198     204       8
     198     258       7     195     198       8     189     195       8     174
     177       6     168     174       7     168     189       7     165     168
       8     153     159       6     153     189       7     147     153       7
     147     165       8     144     147       8     138     144       8     126
     132       6     126     138       7     108     114       3     108     126
       7      99     108       7      81      87       3      81      99       7
      75      81       7      75     138       7      72      75       8      66
      72       8      63      66       8      48      57       6      42      48
       7      42      63       8      12      18       3      12      66       7
       9      12       7       9      33       6       6       9       7       6
      42       7       0       6       6
%FLAG ANGLES_INC_HYDROGEN
%FORMAT(10I8)
     270     264     273       1     267     264     270       1     267     264
     273       1     261     258     264       2     258     264     267       3
     258     264     270       3     258     264     273       3     249     246
     252       5     249     246     258       6     246     252     255       7
     246     258     261       8     237     234     240       5     237     234
     246       6     234     240     243       7     234     246     249       6
     228     222     231       1     225     222     228       1     225     222
     231       1     219     213     222       2     216     213     219      11
     216     213     222       2     213     222     225       3     213     222
     228       3     213     222     231       3     210     207     213       6
     210     207     234       6     207     213     216       8     207     213
     219       8     207     234     237       6     204     207     210      13
     201     198     204      14     201     198     258      15     198     258
     261       8     195     198     201      14     192     189     195      13
     183     174     186      18     177     174     183       5     177     174
     186       5     174     177     180       7     171     168     174       6
     171     168     189       6     168     174     183       6     168     174
     186       6     168     189     192       6     165     168     171      13
     156     153     159       5     156     153     189       6     153     159
     162       7     153     189     192       6     150     147     153      15
     150     147     165      14     147     153     156       6     144     147
     150      14     141     138     144      13     129     126     132       5
     129     126     138       6     126     132     135       7     126     138
     141       6     120     114     123       1     117     114     120       1
```

```
     117     114     123       1     111     108     114       2     111     108
     126       8     108     114     117       3     108     114     120       3
     108     114     123       3     108     126     129       6     105      99
     108      19     102      99     105      20     102      99     108      19
      99     108     111       8      93      87      96       1      90      87
      93       1      90      87      96       1      84      81      87       2
      84      81      99       8      81      87      90       3      81      87
      93       3      81      87      96       3      81      99     102      19
      81      99     105      19      78      75      81       6      78      75
     138       6      75      81      84       8      75     138     141       6
      72      75      78      13      69      66      72      14      63      66
      69      14      54      48      57       5      51      48      54      18
      51      48      57       5      48      57      60       7      45      42
      48       6      45      42      63      13      42      48      51       6
      42      48      54       6      39       6      42       6      30       9
      33       5      24      18      27       1      21      18      24       1
      21      18      27       1      15      12      18       2      15      12
      66       8      12       9      30       6      12      18      21       3
      12      18      24       3      12      18      27       3      12      66
      69      15       9       6      39       6       9      12      15       8
       9      33      36       7       6       9      30       6       6      42
      45       6       3       0       6       7       0       6      39       5
%FLAG ANGLES_WITHOUT_HYDROGEN
%FORMAT(10I8)
     252     246     258       4     246     258     264       9     240     234
     246       4     234     246     252       4     234     246     258      10
     213     207     234      10     207     213     222       9     207     234
     240       4     207     234     246      10     204     198     258      12
     204     207     213      12     204     207     234      12     198     204
     207      16     198     258     246      10     198     258     264       9
     195     198     204      17     195     198     258      12     189     195
     198      16     174     168     189      10     168     174     177       4
     168     189     195      12     165     168     174      12     165     168
     189      12     159     153     189       4     153     147     165      12
     153     189     168      10     153     189     195      12     147     153
     159       4     147     153     189      10     147     165     168      16
     144     147     153      12     144     147     165      17     138     144
     147      16     132     126     138       4     126     138     144      12
     114     108     126       9     108     126     132       4     108     126
     138      10      99     108     114       9      99     108     126      10
      87      81      99       9      81      75     138      10      81      99
     108      10      75      81      87       9      75      81      99      10
      75     138     126      10      75     138     144      12      72      75
      81      12      72      75     138      12      66      72      75      16
      63      66      72      17      48      42      63      12      42      48
      57       4      42      63      66      16      18      12      66       9
      12       9      33       4      12      66      63      12      12      66
      72      12       9       6      42      10       9      12      18       9
       9      12      66      10       6       9      12      10       6       9
      33       4       6      42      48      10       6      42      63      12
       0       6       9       4       0       6      42       4
%FLAG DIHEDRALS_INC_HYDROGEN
%FORMAT(10I8)
     261     258     264     267       1     261     258     264     270       1
     261     258     264     273       1     255     252     246     258       2
     255     252    -246     258       3     252     246     258     261       1
     249     246     252     255       4     249     246     258     261       1
     249     246     258     264       1     246     258     264     267       1
```

246	258	264	270	1	246	258	264	273	1
243	240	234	246	2	243	240	−234	246	3
240	234	246	249	2	240	234	−246	249	5
237	234	240	243	4	237	234	246	249	1
237	234	246	252	2	237	234	−246	252	5
237	234	246	258	1	234	246	252	255	2
234	246	−252	255	3	234	246	258	261	1
219	213	207	234	1	219	213	222	225	1
219	213	222	228	1	219	213	222	231	1
216	213	207	234	1	216	213	222	225	1
216	213	222	228	1	216	213	222	231	1
213	207	234	237	1	210	207	213	216	1
210	207	213	219	1	210	207	213	222	1
210	207	234	237	1	210	207	234	240	2
210	207	−234	240	5	210	207	234	246	1
207	213	222	225	1	207	213	222	228	1
207	213	222	231	1	207	234	240	243	2
207	234	−240	243	3	207	234	246	249	1
204	198	258	261	1	204	207	213	216	1
204	207	213	219	1	204	207	234	237	2
204	207	−234	237	5	201	198	204	207	13
201	198	258	246	1	201	198	258	261	1
201	198	258	264	1	198	204	207	210	13
198	258	246	249	1	198	258	264	267	1
198	258	264	270	1	198	258	264	273	1
195	198	258	261	1	192	189	195	198	13
189	195	198	201	13	186	174	168	189	1
183	174	168	189	1	180	177	174	183	4
180	177	174	186	4	174	168	189	192	1
171	168	174	177	2	171	168	−174	177	5
171	168	174	183	1	171	168	174	186	1
171	168	189	192	1	171	168	189	195	2
171	168	−189	195	5	168	174	177	180	2
168	174	−177	180	3	165	168	174	183	2
165	168	−174	183	5	165	168	174	186	2
165	168	−174	186	5	165	168	189	192	2
165	168	−189	192	5	162	159	153	189	2
162	159	−153	189	3	159	153	189	192	2
159	153	−189	192	5	156	153	147	165	2
156	153	−147	165	5	156	153	159	162	4
156	153	189	168	1	156	153	189	192	1
156	153	189	195	2	156	153	−189	195	5
153	189	168	171	1	150	147	153	156	1
150	147	153	159	1	150	147	153	189	1
150	147	165	168	13	147	153	159	162	2
147	153	−159	162	3	147	153	189	192	1
147	165	168	171	13	144	147	153	156	2
144	147	−153	156	5	141	138	144	147	13
138	144	147	150	13	135	132	126	138	2
135	132	−126	138	3	132	126	138	141	2
132	126	−138	141	5	129	126	132	135	4
129	126	138	141	1	129	126	138	144	2
129	126	−138	144	5	123	114	108	126	1
120	114	108	126	1	117	114	108	126	1
114	108	126	129	1	111	108	114	117	1
111	108	114	120	1	111	108	114	123	1
111	108	126	129	1	111	108	126	132	1
111	108	126	138	1	108	126	132	135	2

```
     108     126    -132     135       3     108     126     138     141       1
     105      99     108     111       1     105      99     108     114       1
     105      99     108     126       3     102      99     108     111       1
     102      99     108     114       1     102      99     108     126       3
      99     108     114     117       1      99     108     114     120       1
      99     108     114     123       1      99     108     126     129       1
      96      87      81      99       1      93      87      81      99       1
      90      87      81      99       1      87      81      99     102       1
      87      81      99     105       1      84      81      75     138       1
      84      81      87      90       1      84      81      87      93       1
      84      81      87      96       1      84      81      99     102       1
      84      81      99     105       1      84      81      99     108       1
      81      75     138     141       1      81      99     108     111       1
      78      75      81      84       1      78      75      81      87       1
      78      75      81      99       1      78      75     138     126       1
      78      75     138     141       1      78      75     138     144       2
      78      75    -138     144       5      75      81      87      90       1
      75      81      87      93       1      75      81      87      96       1
      75      81      99     102       3      75      81      99     105       3
      75     138     126     129       1      72      75      81      84       1
      72      75     138     141       2      72      75    -138     141       5
      69      66      72      75      13      66      72      75      78      13
      54      48      42      63       2      54      48     -42      63       5
      54      48      57      60       4      51      48      42      63       2
      51      48     -42      63       5      51      48      57      60       4
      45      42      48      51       1      45      42      48      54       1
      45      42      48      57       2      45      42     -48      57       5
      45      42      63      66      13      42      48      57      60       2
      42      48     -57      60       3      42      63      66      69      13
      39       6      42      45       1      39       6      42      48       1
      39       6      42      63       2      39       6     -42      63       5
      33       9       6      39       2      33       9      -6      39       5
      30       9       6      39       1      30       9       6      42       1
      30       9      12      66       1      30       9      33      36       4
      27      18      12      66       1      24      18      12      66       1
      21      18      12      66       1      18      12       9      30       1
      18      12      66      69       1      15      12       9      30       1
      15      12       9      33       1      15      12      18      21       1
      15      12      18      24       1      15      12      18      27       1
      15      12      66      63       1      15      12      66      69       1
      15      12      66      72       1      12       9       6      39       1
      12       9      33      36       2      12       9     -33      36       3
       9       6      42      45       1       9      12      18      21       1
       9      12      18      24       1       9      12      18      27       1
       9      12      66      69       1       6       9      12      15       1
       6       9      33      36       2       6       9     -33      36       3
       6      42      48      51       1       6      42      48      54       1
       3       0       6       9       2       3       0      -6       9       3
       3       0       6      39       4       3       0       6      42       2
       3       0      -6      42       3       0       6       9      30       2
       0       6      -9      30       5       0       6      42      45       2
       0       6     -42      45       5
%FLAG DIHEDRALS_WITHOUT_HYDROGEN
%FORMAT(10I8)
     252     246     258     264       1     240     234     246     252       6
     240     234    -246     252       7     240     234     246     258       1
     234     246     258     264       1     222     213     207     234       1
     213     207     234     240       1     213     207     234     246       8
```

213	207	-234	246	9	213	207	-234	246	10
207	204	198	258	11	207	204	-198	258	12
207	234	246	252	1	207	234	-246	258	8
207	234	-246	258	9	207	234	-246	258	10
204	198	258	246	1	204	198	258	264	1
204	207	213	222	1	204	207	234	240	6
204	207	-234	240	7	204	207	-234	246	1
198	204	207	213	11	198	204	-207	213	12
198	204	207	234	11	198	204	-207	234	12
198	258	-246	234	8	198	258	-246	234	9
198	258	-246	234	10	198	258	246	252	1
195	198	204	207	14	195	198	-204	207	15
195	198	-204	207	16	195	198	258	246	1
195	198	258	264	1	189	195	198	204	14
189	195	-198	204	15	189	195	-198	204	16
189	195	198	258	11	189	195	-198	258	12
177	174	168	189	1	174	168	189	195	1
168	189	195	198	11	168	189	-195	198	12
165	147	-153	189	1	165	168	174	177	6
165	168	-174	177	7	165	168	189	195	6
165	168	-189	195	7	159	153	147	165	6
159	153	-147	165	7	159	153	189	168	1
159	153	189	195	6	159	153	-189	195	7
153	147	-165	168	11	153	147	-165	168	12
153	189	-168	165	1	153	189	168	174	8
153	189	-168	174	9	153	189	-168	174	10
153	189	195	198	11	153	189	-195	198	12
147	153	-189	168	8	147	153	-189	168	9
147	153	-189	168	10	147	153	189	195	1
147	165	168	174	11	147	165	-168	174	12
147	165	-168	189	11	147	165	-168	189	12
144	147	153	159	6	144	147	-153	159	7
144	147	153	189	1	144	147	165	168	14
144	147	-165	168	15	144	147	-165	168	16
138	144	147	153	11	138	144	-147	153	12
138	144	147	165	14	138	144	-147	165	15
138	144	-147	165	16	132	126	138	144	6
132	126	-138	144	7	126	138	144	147	11
126	138	-144	147	12	114	108	126	132	1
114	108	126	138	1	108	126	138	144	1
99	81	75	138	8	99	81	-75	138	9
99	81	-75	138	10	99	108	126	132	1
99	108	-126	138	8	99	108	-126	138	9
99	108	-126	138	10	87	81	75	138	1
87	81	99	108	1	81	75	138	126	8
81	75	-138	126	9	81	75	-138	126	10
81	75	138	144	1	81	99	108	114	1
81	99	-108	126	8	81	99	-108	126	9
81	99	-108	126	10	75	81	99	108	8
75	81	-99	108	9	75	81	-99	108	10
75	138	-126	108	8	75	138	-126	108	9
75	138	-126	108	10	75	138	126	132	1
75	138	144	147	11	75	138	-144	147	12
72	75	81	87	1	72	75	81	99	1
72	75	138	126	1	72	75	138	144	6
72	75	-138	144	7	66	72	75	81	11
66	72	-75	81	12	66	72	75	138	11
66	72	-75	138	12	63	66	72	75	14

```
      63      66     -72      75      15      63      66     -72      75      16
      57      48      42      63       6      57      48     -42      63       7
      48      42      63      66      11      48      42     -63      66      12
      42      63      66      72      14      42      63     -66      72      15
      42      63     -66      72      16      33       9       6      42       1
      33       9      12      66       1      18      12       9      33       1
      18      12      66      63       1      18      12      66      72       1
      12       9       6      42       8      12       9      -6      42       9
      12       9      -6      42      10      12      66     -63      42      11
      12      66     -63      42      12      12      66      72      75      11
      12      66     -72      75      12       9       6      42      48       8
       9       6     -42      48       9       9       6     -42      48      10
       9       6      42      63       1       9      12     -66      63       1
       9      12      66      72       1       6       9      12      18       1
       6       9      12      66       8       6       9     -12      66       9
       6       9     -12      66      10       6      42      48      57       1
       6      42     -63      66      11       6      42     -63      66      12
       0       6       9      12       1       0       6       9      33       6
       0       6      -9      33       7       0       6      42      48       1
       0       6      42      63       6       0       6     -42      63       7
%FLAG EXCLUDED_ATOMS_LIST
%FORMAT(10I8)
       2       3       4       5      11      12      14      15      16      17
      22       3       4      14      15       4       5       6       7      11
      12      13      14      15      16      17      18      19      20      22
      23       5       6       7       8       9      10      11      12      13
      14      15      16      17      22      23      24      25       6       7
       8       9      10      11      12      13      14      15      22      23
      24      25      26       7       8       9      10      11      12      22
      23      24      25       8       9      10      11      12      22      23
      24      25       9      10      23      10      23      23      12      13
      14      15      23      13      14      15      23       0      15      16
      17      22      16      17      18      19      20      21      22      23
      24      25      17      18      19      20      22      23      18      19
      20      21      22      23      19      20      21      22      20      21
      22      21      22       0      23      24      25      26      24      25
      26      27      28      47      25      26      26      27      28      29
      30      34      43      47      48      49      27      28      29      30
      31      32      33      34      35      36      37      43      44      45
      47      48      49      50      28      29      30      34      43      47
      48      49      29      30      31      32      33      34      35      36
      37      38      39      43      47      48      49      30      31      32
      33      34      35      36      37      47      31      32      33      34
      35      36      37      47      32      33      34      33      34      34
      35      36      37      38      39      40      41      42      43      44
      45      47      36      37      38      39      43      37      38      39
      43      38      39      40      41      42      43      44      45      46
      47      48      49      39      40      41      42      43      44      45
      47      40      41      42      43      44      45      47      41      42
      43      42      43      43      44      45      46      47      48      49
      50      45      46      47      48      49      46      47      48      49
      47      48      49      50      51      52      56      49      50      50
      51      52      53      54      56      57      64      51      52      53
      54      55      56      57      58      59      64      65      66      52
      53      54      56      57      64      53      54      55      56      57
      58      59      64      65      66      67      54      55      56      57
      64      65      66      55      56      57      64      65      66      64
      57      58      59      60      62      63      64      65      66      58
```

```
      59      60      61      62      63      64      65      66      67      59
      60      62      63      64      65      66      60      61      62      63
      64      65      66      61      62      63      64      62      63      63
      64      64      65      66      67      68      69      87      66      67
      67      68      69      70      83      87      88      89      68      69
      70      71      72      79      83      84      85      87      88      89
      90      91      92      69      70      83      87      88      89      70
      71      72      73      74      75      79      80      81      83      87
      88      89      71      72      73      74      75      76      77      78
      79      80      81      82      83      84      85      87      72      73
      74      75      79      80      81      83      73      74      75      76
      77      78      79      80      81      83      74      75      76      77
      78      79      75      76      77      78      79      76      77      78
      79      77      78      78       0      80      81      82      83      84
      85      86      87      88      89      81      82      83      84      85
      87      82      83      84      85      87      83      84      85      86
      87      88      89      90      91      92      85      86      87      88
      89      86      87      88      89      87      88      89      90      91
      92      89      90      91      92      90      91      92      91      92
      92       0
%FLAG HBOND_ACOEF
%FORMAT(5E16.8)

%FLAG HBOND_BCOEF
%FORMAT(5E16.8)

%FLAG HBCUT
%FORMAT(5E16.8)

%FLAG AMBER_ATOM_TYPE
%FORMAT(20a4)
oh  ho  c3  c3  c3  hx  n4  hn  hn  hn  h1  oh  ho  h1  c3  h1  c3  h1  h1  oh
ho  os  c3  h2  os  c3  h1  c3  hx  n4  hn  hn  hn  c3  hc  hc  c3  hx  n4  hn
hn  hn  c3  h1  oh  ho  c3  h1  os  c3  h2  c3  h1  oh  ho  os  c3  h1  c3  oh
ho  h1  h1  c3  h1  os  c3  h2  os  c3  h1  c3  hx  hx  n4  hn  hn  hn  c3  h1
oh  ho  c3  h1  oh  ho  c3  hx  n4  hn  hn  hn
%FLAG TREE_CHAIN_CLASSIFICATION
%FORMAT(20a4)
M   E   M   M   M   E   3   E   E   E   E   S   E   E   3   E   3   E   E   S
E   E   M   E   M   M   E   3   E   3   E   E   E   3   E   E   3   E   3   E
E   E   B   E   S   E   M   E   M   M   E   M   E   S   E   S   B   E   3   S
E   E   E   M   E   M   M   E   S   3   E   3   E   E   3   E   E   E   3   E
S   E   B   E   S   E   M   E   M   E   E   E
%FLAG JOIN_ARRAY
%FORMAT(10I8)
       0       0       0       0       0       0       0       0       0       0
       0       0       0       0       0       0       0       0       0       0
       0       0       0       0       0       0       0       0       0       0
       0       0       0       0       0       0       0       0       0       0
       0       0       0       0       0       0       0       0       0       0
       0       0       0       0       0       0       0       0       0       0
       0       0       0       0       0       0       0       0       0       0
       0       0       0       0       0       0       0       0       0       0
       0       0       0       0       0       0       0       0       0       0
       0       0
%FLAG IROTAT
%FORMAT(10I8)
       0       0       0       0       0       0       0       0       0       0
```

```
       0       0       0       0       0       0       0       0       0       0
       0       0       0       0       0       0       0       0       0       0
       0       0       0       0       0       0       0       0       0       0
       0       0       0       0       0       0       0       0       0       0
       0       0       0       0       0       0       0       0       0       0
       0       0       0       0       0       0       0       0       0       0
       0       0       0       0       0       0       0       0       0       0
       0       0       0       0       0       0       0       0       0       0
       0       0
%FLAG RADIUS_SET
%FORMAT(1a80)
modified Bondi radii (mbondi)
%FLAG RADII
%FORMAT(5E16.8)
  1.50000000E+00  8.00000000E-01  1.70000000E+00  1.70000000E+00  1.70000000E+00
  1.30000000E+00  1.55000000E+00  1.30000000E+00  1.30000000E+00  1.30000000E+00
  1.30000000E+00  1.50000000E+00  8.00000000E-01  1.30000000E+00  1.70000000E+00
  1.30000000E+00  1.70000000E+00  1.30000000E+00  1.30000000E+00  1.50000000E+00
  8.00000000E-01  1.50000000E+00  1.70000000E+00  1.30000000E+00  1.50000000E+00
  1.70000000E+00  1.30000000E+00  1.70000000E+00  1.30000000E+00  1.55000000E+00
  1.30000000E+00  1.30000000E+00  1.30000000E+00  1.70000000E+00  1.30000000E+00
  1.30000000E+00  1.70000000E+00  1.30000000E+00  1.55000000E+00  1.30000000E+00
  1.30000000E+00  1.30000000E+00  1.70000000E+00  1.30000000E+00  1.50000000E+00
  8.00000000E-01  1.70000000E+00  1.30000000E+00  1.50000000E+00  1.70000000E+00
  1.30000000E+00  1.70000000E+00  1.30000000E+00  1.50000000E+00  8.00000000E-01
  1.50000000E+00  1.70000000E+00  1.30000000E+00  1.70000000E+00  1.50000000E+00
  8.00000000E-01  1.30000000E+00  1.30000000E+00  1.70000000E+00  1.30000000E+00
  1.50000000E+00  1.70000000E+00  1.30000000E+00  1.50000000E+00  1.70000000E+00
  1.30000000E+00  1.70000000E+00  1.30000000E+00  1.30000000E+00  1.55000000E+00
  1.30000000E+00  1.30000000E+00  1.30000000E+00  1.70000000E+00  1.30000000E+00
  1.50000000E+00  8.00000000E-01  1.70000000E+00  1.30000000E+00  1.50000000E+00
  8.00000000E-01  1.70000000E+00  1.30000000E+00  1.55000000E+00  1.30000000E+00
  1.30000000E+00  1.30000000E+00
%FLAG SCREEN
%FORMAT(5E16.8)
  8.50000000E-01  8.50000000E-01  7.20000000E-01  7.20000000E-01  7.20000000E-01
  8.50000000E-01  7.90000000E-01  8.50000000E-01  8.50000000E-01  8.50000000E-01
  8.50000000E-01  8.50000000E-01  8.50000000E-01  8.50000000E-01  7.20000000E-01
  8.50000000E-01  7.20000000E-01  8.50000000E-01  8.50000000E-01  8.50000000E-01
  8.50000000E-01  8.50000000E-01  7.20000000E-01  8.50000000E-01  8.50000000E-01
  7.20000000E-01  8.50000000E-01  7.20000000E-01  8.50000000E-01  7.90000000E-01
  8.50000000E-01  8.50000000E-01  8.50000000E-01  7.20000000E-01  8.50000000E-01
  8.50000000E-01  7.20000000E-01  8.50000000E-01  7.90000000E-01  8.50000000E-01
  8.50000000E-01  8.50000000E-01  7.20000000E-01  8.50000000E-01  8.50000000E-01
  8.50000000E-01  7.20000000E-01  8.50000000E-01  8.50000000E-01  7.20000000E-01
  8.50000000E-01  7.20000000E-01  8.50000000E-01  8.50000000E-01  8.50000000E-01
  8.50000000E-01  7.20000000E-01  8.50000000E-01  7.20000000E-01  8.50000000E-01
  8.50000000E-01  8.50000000E-01  8.50000000E-01  7.20000000E-01  8.50000000E-01
  8.50000000E-01  7.20000000E-01  8.50000000E-01  8.50000000E-01  7.20000000E-01
  8.50000000E-01  7.20000000E-01  8.50000000E-01  8.50000000E-01  7.90000000E-01
  8.50000000E-01  8.50000000E-01  8.50000000E-01  7.20000000E-01  8.50000000E-01
  8.50000000E-01  8.50000000E-01  7.20000000E-01  8.50000000E-01  8.50000000E-01
  8.50000000E-01  7.20000000E-01  8.50000000E-01  7.90000000E-01  8.50000000E-01
  8.50000000E-01  8.50000000E-01
```
