# Supplementary material for: Understanding the Origins of Bacterial Resistance to Aminoglycosides through Molecular Dynamics Mutational Study of the Ribosomal A-Site
Source: PLoS Comput Biol. 2011 Jul 21;7(7):e1002099. doi: 10.1371/journal.pcbi.1002099 (PMC3140962; doi:10.1371/journal.pcbi.1002099)
Supplement: Figure S1 — Distances between the atoms of paromomycin and RNA bases [Å] in the NON_MUT_PAR simulation and in the X-ray structure (PDB code: 1J7T) [Vicens, Q.; Westhof, E. Structure . 2001, 9, 647–58]. (PDF) [file pcbi.1002099.s002.pdf]

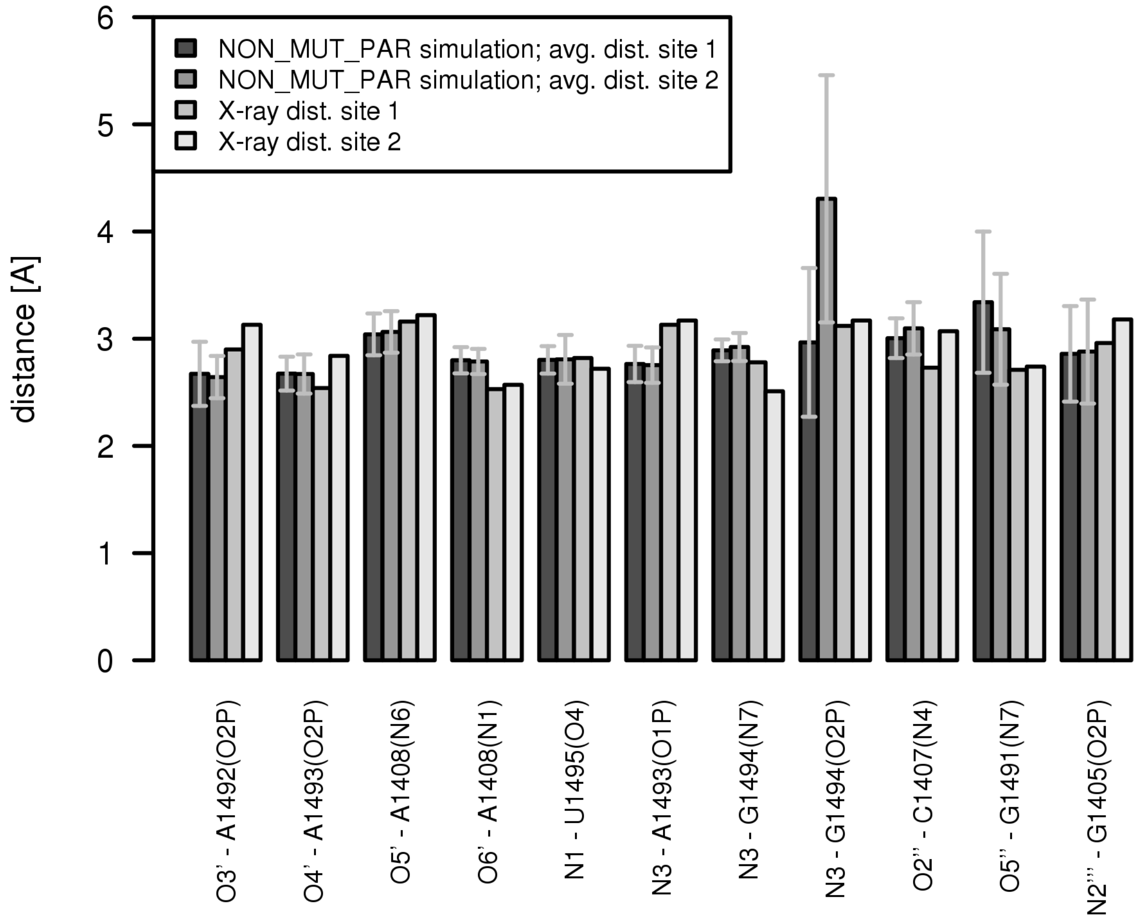

Figure S1: **Distances between the atoms of paromomycin and RNA bases [Å]** in the NON_MUT_PAR simulation and in the X-ray structure (PDB code: 1J7T) [Vicens, Q.; Westhof, E. *Structure*. **2001**, 9, 647-58].
