# Supplementary material for: Understanding the Origins of Bacterial Resistance to Aminoglycosides through Molecular Dynamics Mutational Study of the Ribosomal A-Site
Source: PLoS Comput Biol. 2011 Jul 21;7(7):e1002099. doi: 10.1371/journal.pcbi.1002099 (PMC3140962; doi:10.1371/journal.pcbi.1002099)
Supplement: Figure S2 — Average RMSD [Ångstrom] with their standard deviations; two graphs depict RMSD for two A-sites. (PDF) [file pcbi.1002099.s003.pdf]

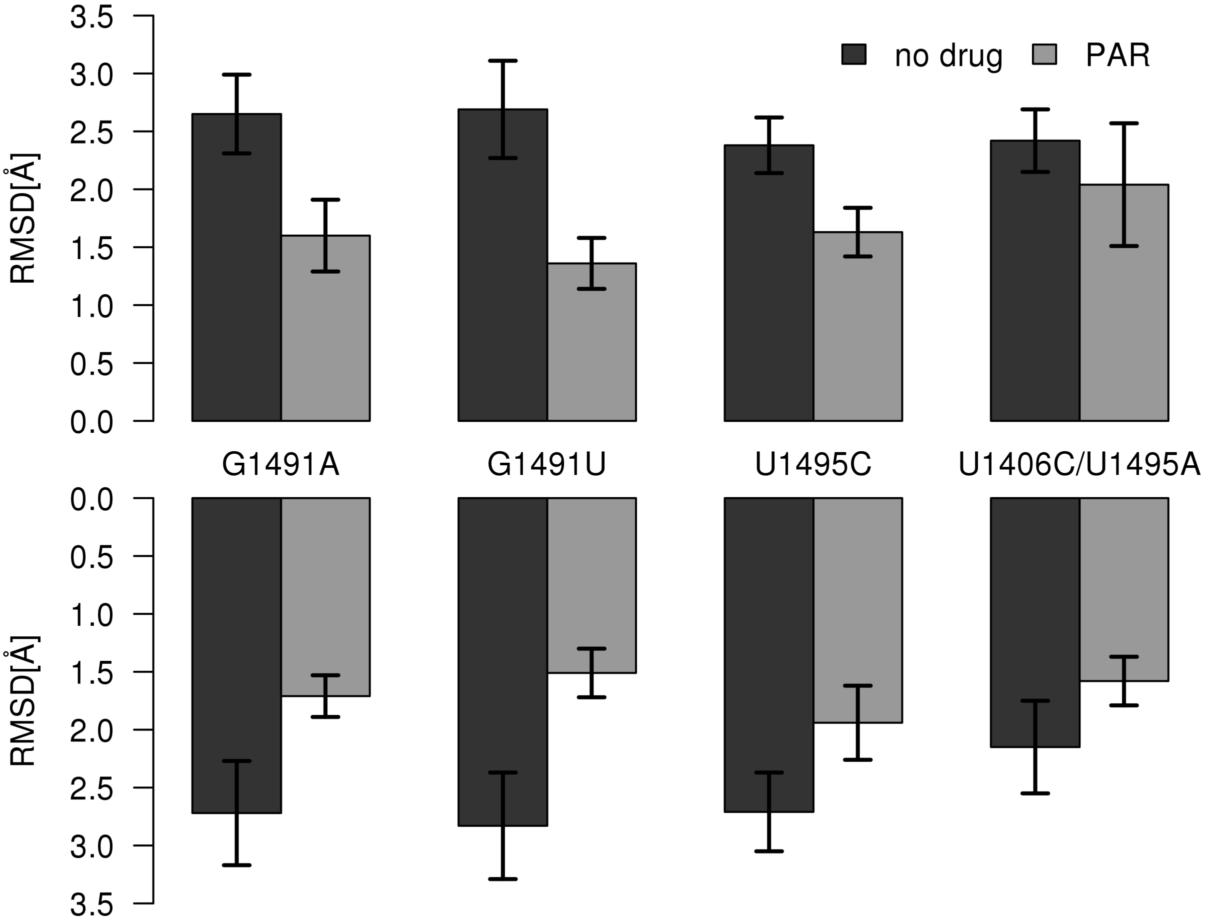

Figure S2: **Average RMSD [Ångstrom]** with their standard deviations; two graphs depict RMSD for two A-sites.
