# Supplementary material for: Understanding the Origins of Bacterial Resistance to Aminoglycosides through Molecular Dynamics Mutational Study of the Ribosomal A-Site
Source: PLoS Comput Biol. 2011 Jul 21;7(7):e1002099. doi: 10.1371/journal.pcbi.1002099 (PMC3140962; doi:10.1371/journal.pcbi.1002099)
Supplement: Figure S3 — Pseudo-dihedral angles describing the variation in the conformations of A1492 and A1493. The angle is calculated as a torsion angle between the four atoms: G1494(N1) – G1494(P) – A1493(P) – A1493(N1), as depicted by black sticks (analogously for : G1494(N1) – G1494(P) – A1492(P) – A1492(N1)). The exemplary snapshot is taken from the NON_MUT simulation, with the values of the pseudo-dihedral angles (in degrees) shown in brackets. (PDF) [file pcbi.1002099.s004.pdf]

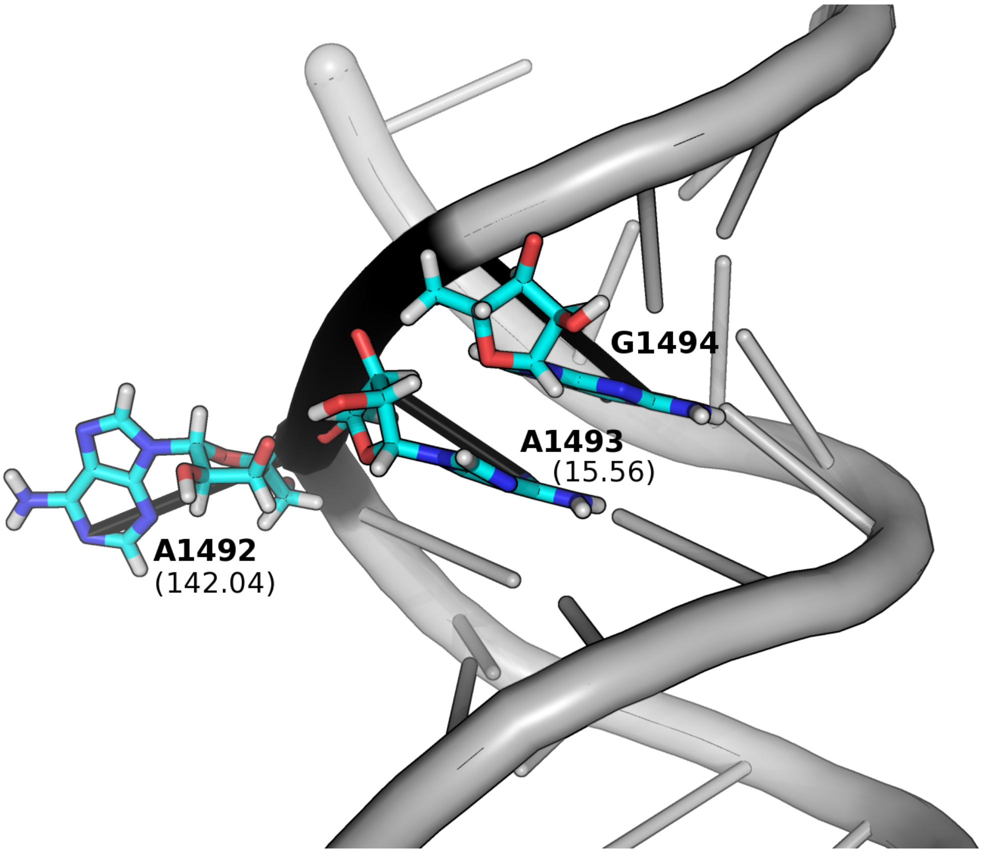


Figure S3: **Pseudo-dihedral angles describing the variation in the conformations of A1492 and A1493**. The angle $\theta_{A1493}$ is calculated as a torsion angle between the four atoms: G1494(N1) – G1494(P) – A1493(P) – A1493(N1), as depicted by black sticks (analogously for $\theta_{A1492}$: G1494(N1) – G1494(P) – A1492(P) – A1492(N1)). The exemplary snapshot is taken from the NON_MUT simulation, with the values of the pseudo-dihedral angles (in degrees) shown in brackets.
