# Supplementary material for: Understanding the Origins of Bacterial Resistance to Aminoglycosides through Molecular Dynamics Mutational Study of the Ribosomal A-Site
Source: PLoS Comput Biol. 2011 Jul 21;7(7):e1002099. doi: 10.1371/journal.pcbi.1002099 (PMC3140962; doi:10.1371/journal.pcbi.1002099)
Supplement: Figure S4 — Conformations of bases forming the A-site in the most populated clusters in different MD simulations. The distances (in Å) between chosen atoms are shown as black dotted lines; two snapshots per simulation correspond to two A-sites in the crystal structure; for base numbering see the inset in the Figure S5 and Figure 1a. (PDF) [file pcbi.1002099.s005.pdf]

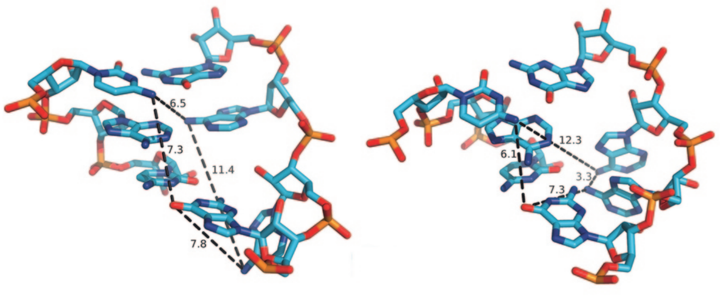

(a) NON_MUT

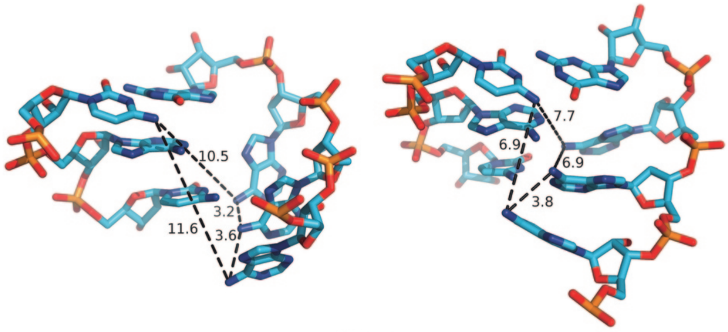

(b) G1491A

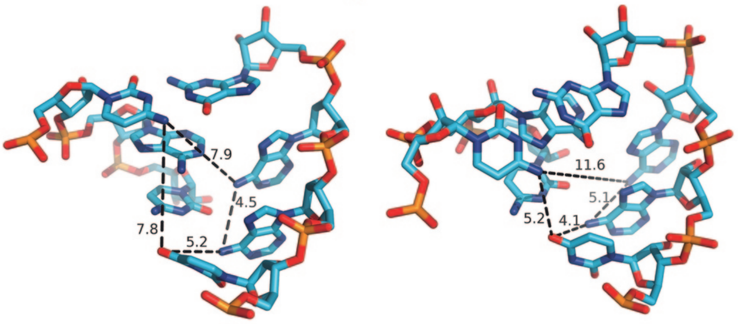

(c) G1491U

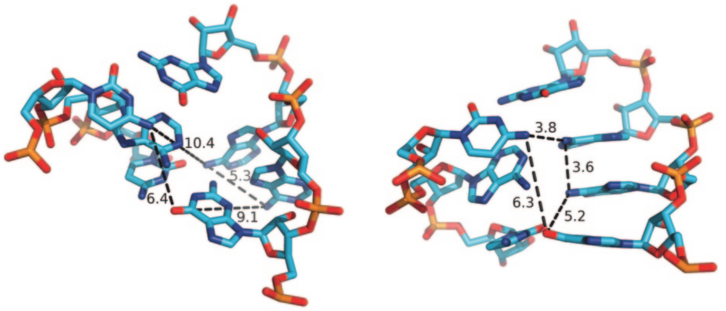

(d) U1495C

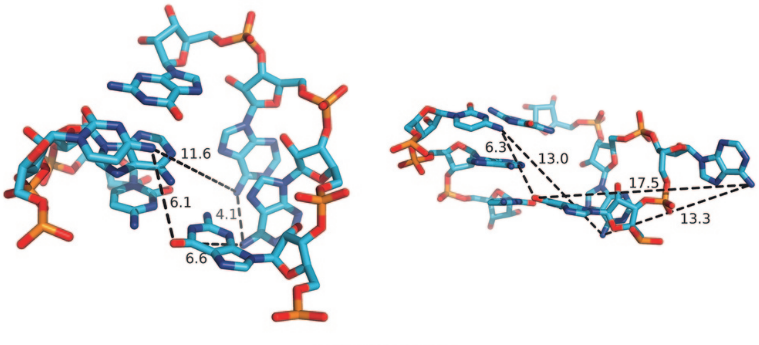

(d) U1406C/U1495A

Figure S4: **Conformations of bases forming the A-site in the most populated clusters in different MD simulations**. The distances (in Å) between chosen atoms are shown as black dotted lines; two snapshots per simulation correspond to two A-sites in the crystal structure; for base numbering see the inset in the Figure S5 and Figure 1a.
