# Supplementary material for: Understanding the Origins of Bacterial Resistance to Aminoglycosides through Molecular Dynamics Mutational Study of the Ribosomal A-Site
Source: PLoS Comput Biol. 2011 Jul 21;7(7):e1002099. doi: 10.1371/journal.pcbi.1002099 (PMC3140962; doi:10.1371/journal.pcbi.1002099)
Supplement: Figure S5 — Distances between chosen atoms inside the A-site as a function of the simulation time. Four sets of graphs correspond to four distances (1, 2, 3, 4) depicted as black dotted lines on a stick model of the A-site fragment; for base numbering see Figure 1a. Grey and black lines are for two simulated A-sites. (PDF) [file pcbi.1002099.s006.pdf]

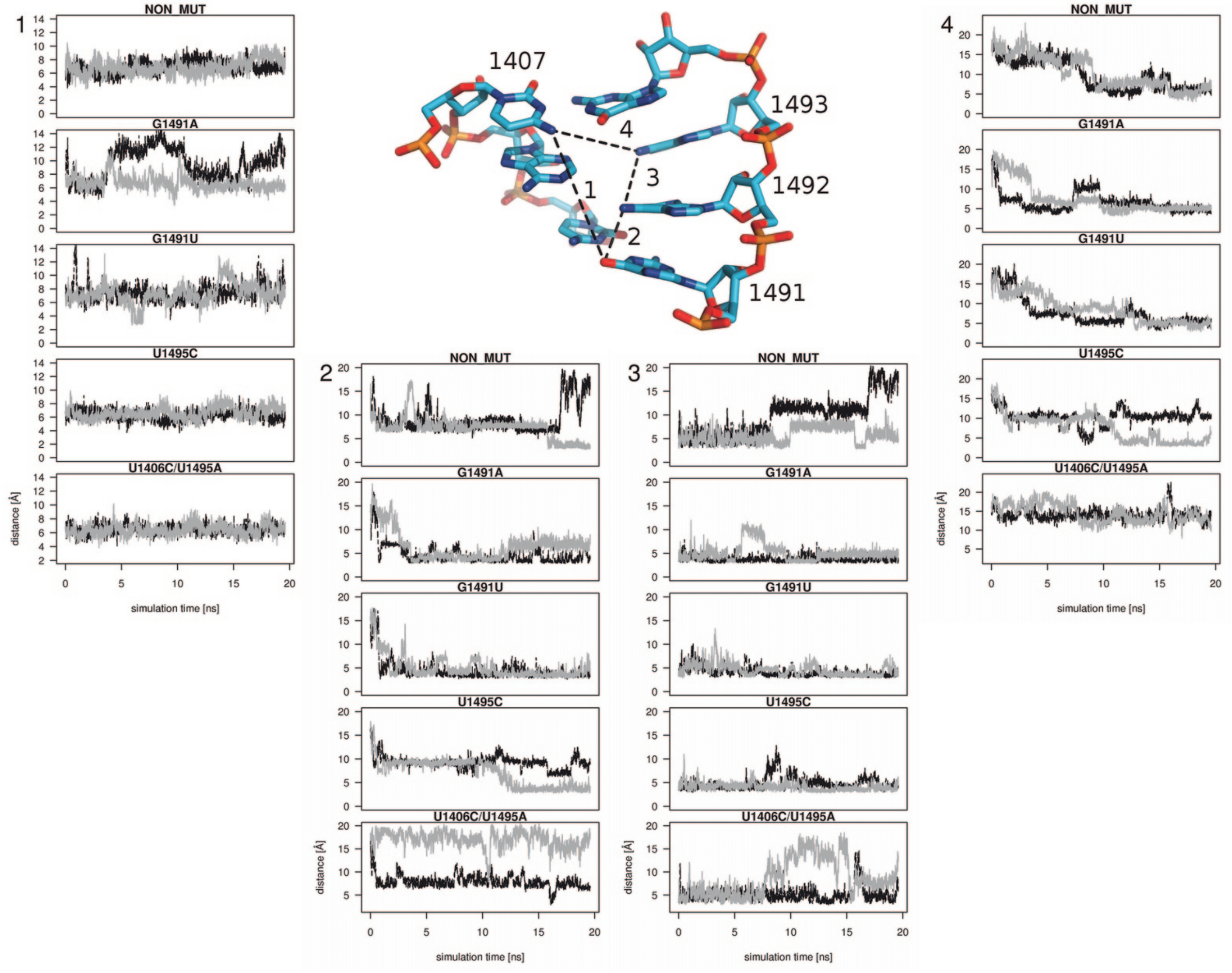

Figure S5: **Distances between chosen atoms inside the A-site as a function of the simulation time.** Four sets of graphs correspond to four distances (1, 2, 3, 4) depicted as black dotted lines on a stick model of the A-site fragment; for base numbering see Figure 1a. Grey and black lines are for two simulated A-sites.
