# Supplementary material for: Understanding the Origins of Bacterial Resistance to Aminoglycosides through Molecular Dynamics Mutational Study of the Ribosomal A-Site
Source: PLoS Comput Biol. 2011 Jul 21;7(7):e1002099. doi: 10.1371/journal.pcbi.1002099 (PMC3140962; doi:10.1371/journal.pcbi.1002099)
Supplement: Figure S7 — Representative structures of the clusters for different MD simulations of the A-site in the complex with paromomycin, superposed with regard to phosphorous atoms. Structures from each cluster are colored differently: 1 – green, 2 – blue, 3 – red, 4 – yellow, 5 – cyan (see Table S1 for the cluster sizes). Paromomycin and A1492, A1493 and A1408 are shown in atomic detail. (PDF) [file pcbi.1002099.s008.pdf]

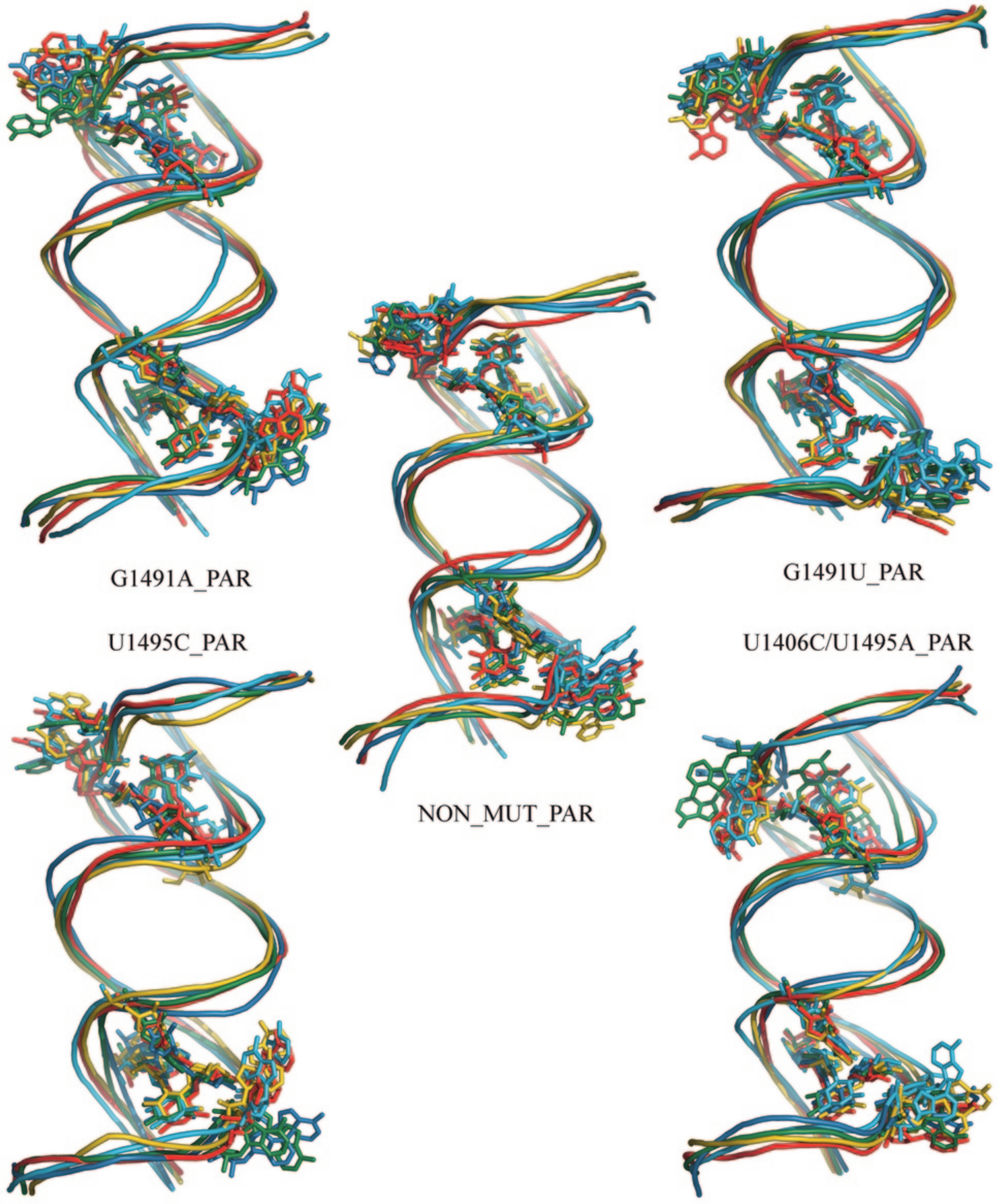

Figure S7: **Representative structures of the clusters for different MD simulations of the A-site in the complex with paromomycin**, superposed with regard to phosphorous atoms. Structures from each cluster are colored differently: 1 — green, 2 — blue, 3 — red, 4 — yellow, 5 — cyan (see Table S1 for the cluster sizes). Paromomycin and A1492, A1493 and A1408 are shown in atomic detail.
