# Supplementary material for: Understanding the Origins of Bacterial Resistance to Aminoglycosides through Molecular Dynamics Mutational Study of the Ribosomal A-Site
Source: PLoS Comput Biol. 2011 Jul 21;7(7):e1002099. doi: 10.1371/journal.pcbi.1002099 (PMC3140962; doi:10.1371/journal.pcbi.1002099)
Supplement: Figure S8 — Distances between the centers of mass of adenine phosphorous atoms, A1492(P) and A1493(P), and paromomycin ring III ( left ) or ring IV ( right ). The frequency distributions of the distances are shown next to each graph. Black and grey lines correspond to the two A-sites of the crystal structure. (PDF) [file pcbi.1002099.s009.pdf]

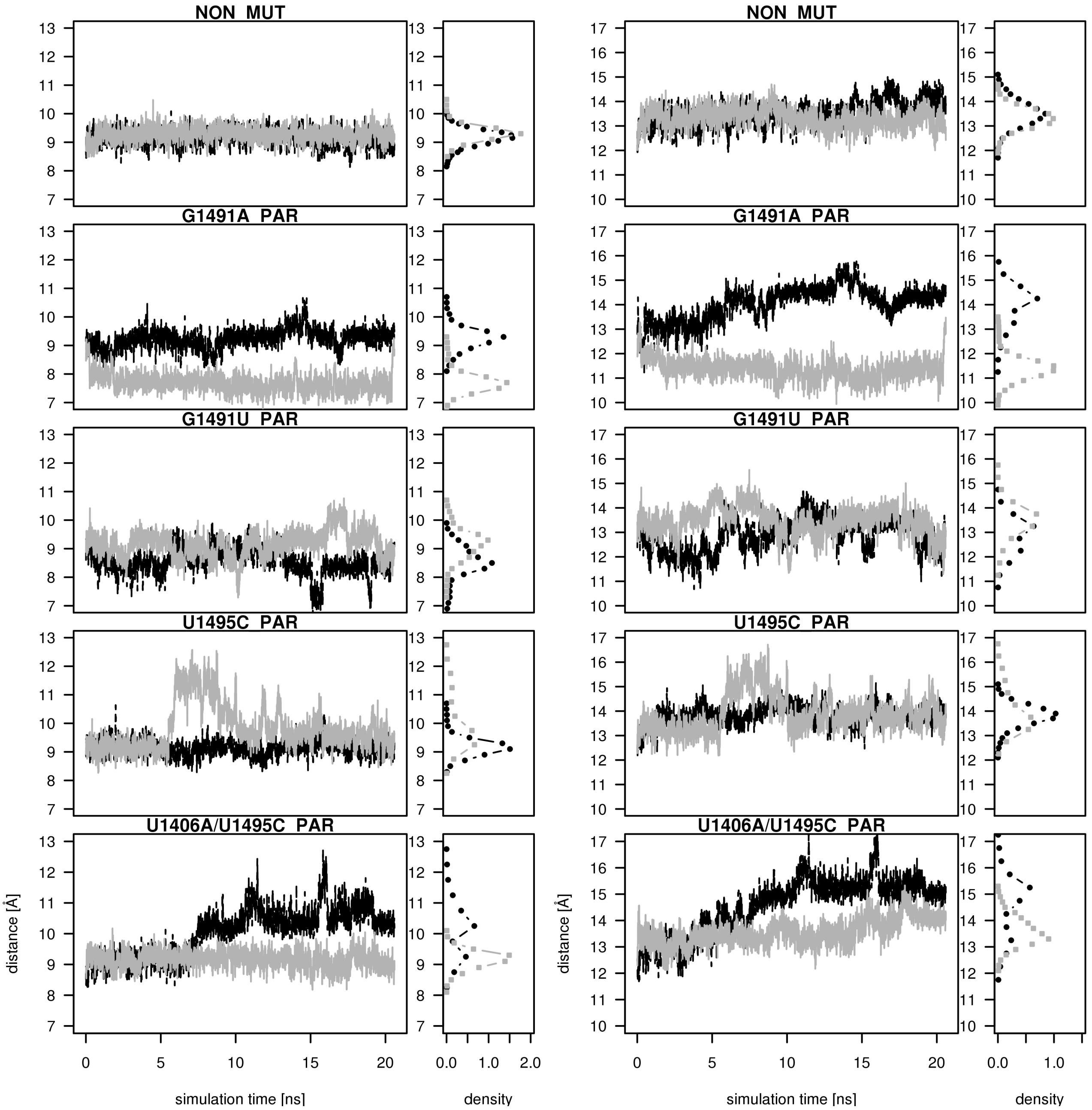

Figure S8: **Distances between the centers of mass of adenine phosphorous atoms, A1492(P) and A1493(P), and paromomycin ring III (*left*) or ring IV (*right*).** The frequency distributions of the distances are shown next to each graph. Black and grey lines correspond to the two A-sites of the crystal structure.
