# Supplementary material for: Understanding the Origins of Bacterial Resistance to Aminoglycosides through Molecular Dynamics Mutational Study of the Ribosomal A-Site
Source: PLoS Comput Biol. 2011 Jul 21;7(7):e1002099. doi: 10.1371/journal.pcbi.1002099 (PMC3140962; doi:10.1371/journal.pcbi.1002099)
Supplement: Figure S10 — Areas of high water density (light blue, 0.23 water oxygens per ) located in one part of the simulated structure, superposed on the crystal structure of the complex with paromomycin (PDB entry 1J7T). Only the U1406·U1495 and A/G1408, A1492, A1493 bases are shown in atomic details; spheres show the positions of the crystal water oxygen atoms, the ones which were identified in the simulation are marked in orange (see also Table S3). Hydrogen atoms were not shown for clarity of the image. (PDF) [file pcbi.1002099.s011.pdf]

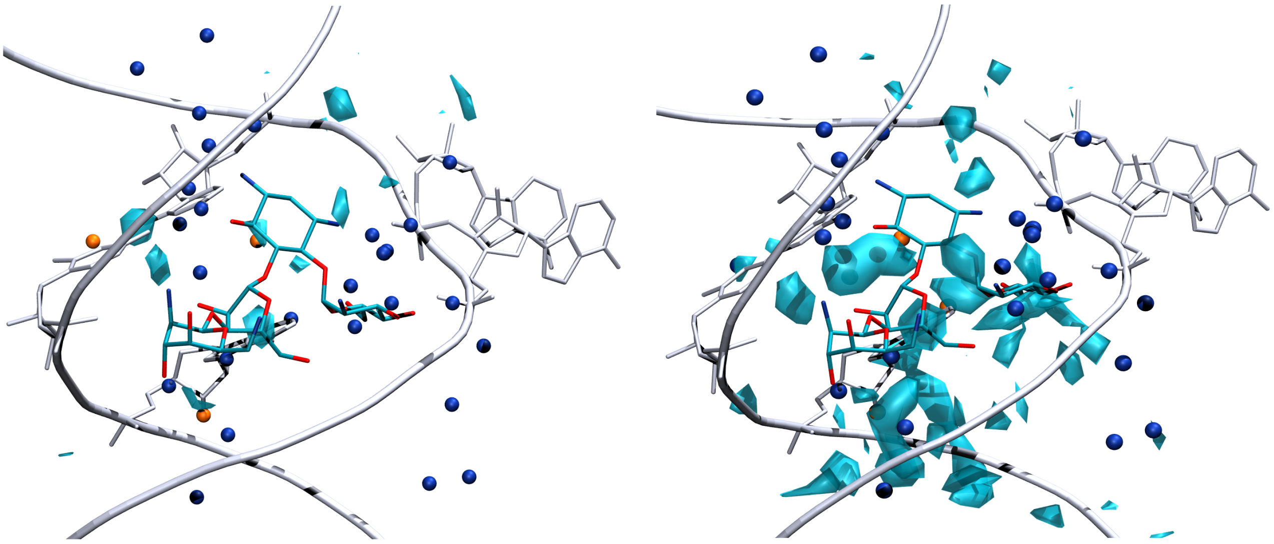

(a) G1491A

(b) U1406C/U1495A

Figure S10: **Areas of high water density** (light blue, $\geq$ 0.23 water oxygens per Å^3) located in one part of the simulated structure, superposed on the crystal structure of the complex with paromomycin (PDB entry 1J7T); only the U1406○U1495 and A/G1408, A1492, A1493 bases are shown in atomic details; spheres show the positions of the crystal water oxygen atoms, the ones which were identified in the simulation are marked in orange (see also Table S3). Hydrogen atoms were not shown for clarity of the image.
