# Supplementary material for: Understanding the Origins of Bacterial Resistance to Aminoglycosides through Molecular Dynamics Mutational Study of the Ribosomal A-Site
Source: PLoS Comput Biol. 2011 Jul 21;7(7):e1002099. doi: 10.1371/journal.pcbi.1002099 (PMC3140962; doi:10.1371/journal.pcbi.1002099)
Supplement: Figure S11 — Solvent accessible surface area (SASA) [ ] of the two adenines A1492 and A1493, calculated for the ribosome structures available in PDB in higher resolution (i.e., structures containing only phosphorous atoms were discarded). The values are compared with an average and standard deviation calculated for the NON_MUT simulation (for both symmetrical parts of the model structure, without taking into account the hydrogen atoms, since X-ray structures do not contain these). (PDF) [file pcbi.1002099.s012.pdf]

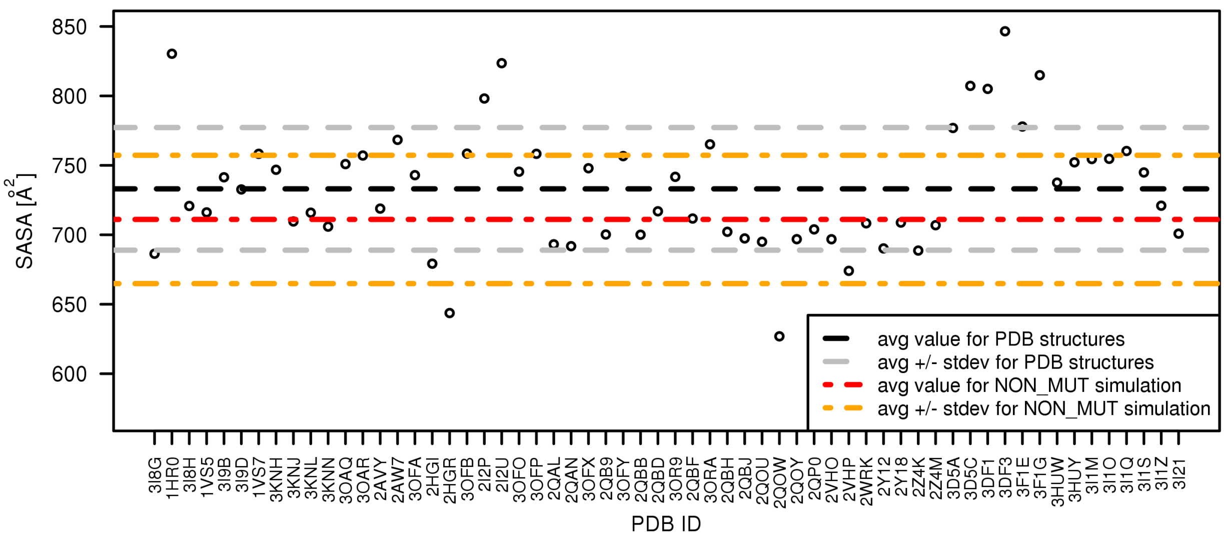

Figure S11: **Solvent accessible surface area (SASA) [Å^2] of the two adenines A1492 and A1493**, calculated for the ribosome structures available in PDB in higher resolution (i.e., structures containing only phosphorous atoms were discarded). The values are compared with an avarage and standard deviation calculated for the NON_MUT simulation (for both symmetrical parts of the model structure, without taking into account the hydrogen atoms, since X-ray structures do not contain these).
