# Supplementary material for: Understanding the Origins of Bacterial Resistance to Aminoglycosides through Molecular Dynamics Mutational Study of the Ribosomal A-Site
Source: PLoS Comput Biol. 2011 Jul 21;7(7):e1002099. doi: 10.1371/journal.pcbi.1002099 (PMC3140962; doi:10.1371/journal.pcbi.1002099)
Supplement: Figure S12 — Average glycosidic torsion angle , calculated for all the residues in each simulation per each frame. The scale on y axes spans the possible range of (−180, +180); compare with Figure 6 in [Banáš, P. et al., Journal of Chemical Theory and Computation. 2010, 6, 3836–3849]. (PDF) [file pcbi.1002099.s013.pdf]

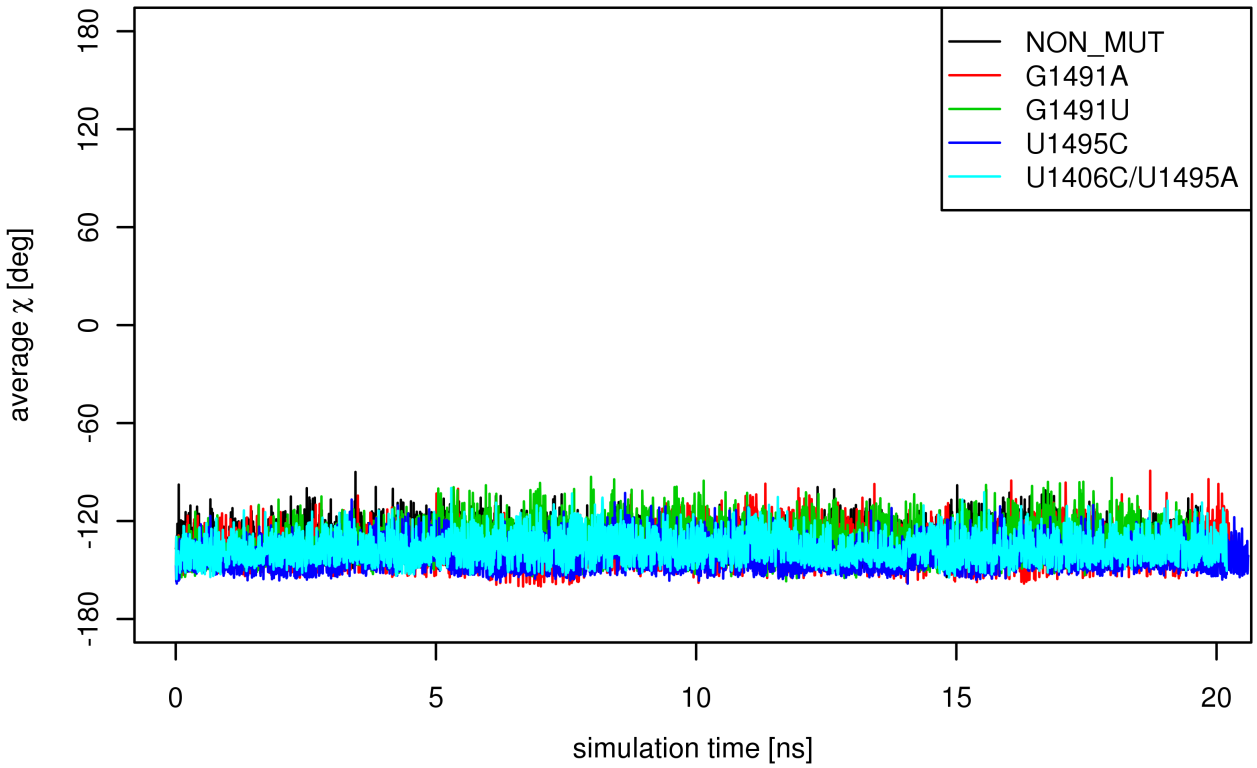

Figure S12: **Average glycosidic torsion angle** χ, calculated for all the residues in each simulation per each frame. The scale on y axes spans the possible range of χ (-180, +180); compare with Figure 6 in [Banáš, P. *et al.*, Journal of Chemical Theory and Computation. **2010**, 6, 3836-3849].
