# Supplementary material for: Understanding the Origins of Bacterial Resistance to Aminoglycosides through Molecular Dynamics Mutational Study of the Ribosomal A-Site
Source: PLoS Comput Biol. 2011 Jul 21;7(7):e1002099. doi: 10.1371/journal.pcbi.1002099 (PMC3140962; doi:10.1371/journal.pcbi.1002099)
Supplement: Figure S13 — Dihedral angles of linkages between the rings of paromomycin. The angles and are defined as in [Asensio, J. L. et al., Chemistry. 2002, 8, 5228–40], an NMR study of neomycin, which differs from paromomycin only with one chemical group, having OH instead of . The values were collected during two independent 10-ns production phases of MD of paromomycin in water (the two runs started from the same minimized structure but with different initial velocities). (PDF) [file pcbi.1002099.s014.pdf]

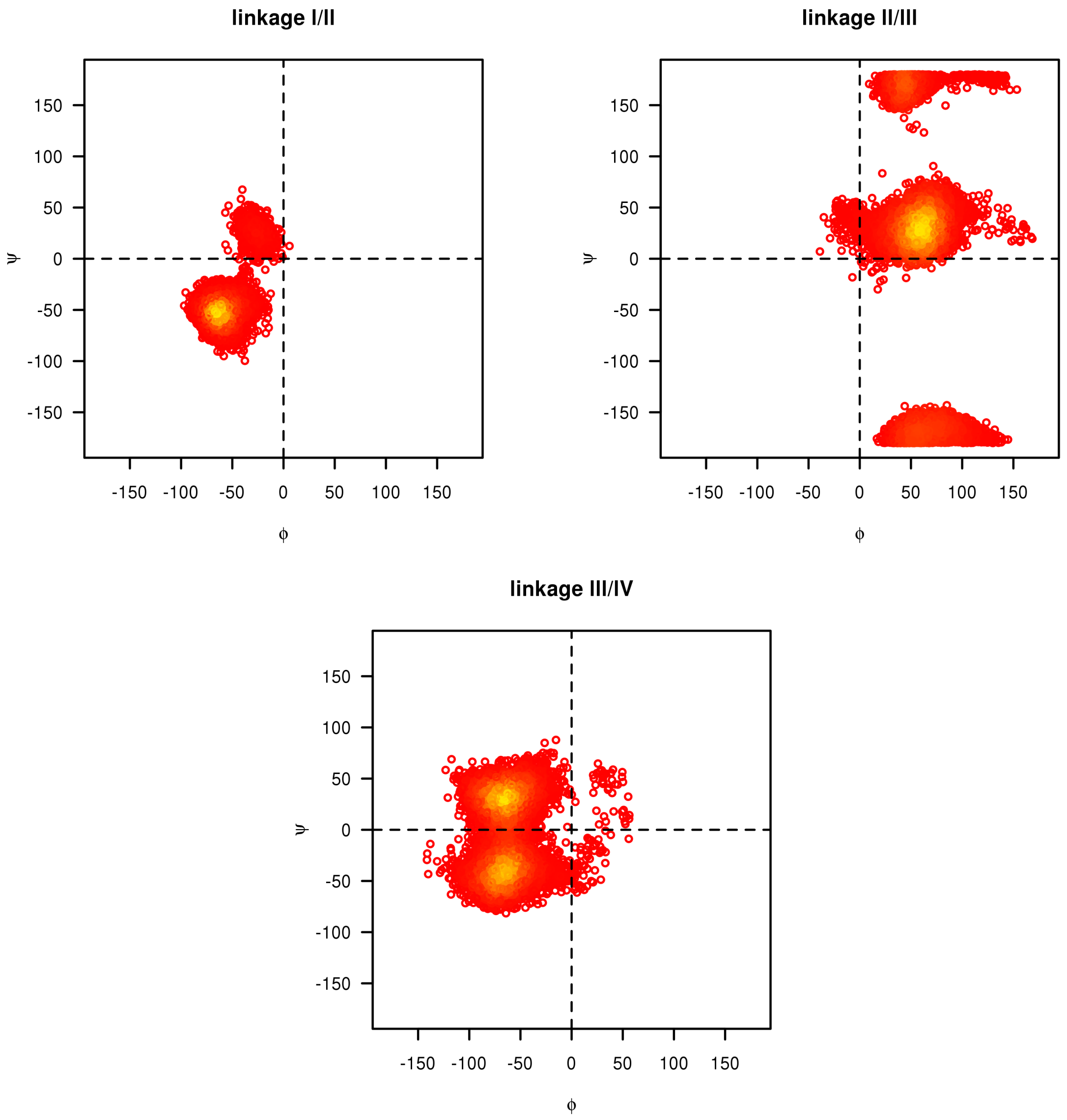

Figure S13: **Dihedral angles of linkages between the rings of paromomycin.** The angles ϕ and ψ are defined as in [Asensio, J. L. *et al.*, Chemistry. **2002**, 8, 5228-40], an NMR study of neomycin, which differs from paromomycin only with one chemical group, having OH instead of NH_3^+. The values were collected during two independent 10-ns production phases of MD of paromomycin in water (the two runs started from the same minimized structure but with different initial velocities).
