# Supplementary material for: Understanding the Origins of Bacterial Resistance to Aminoglycosides through Molecular Dynamics Mutational Study of the Ribosomal A-Site
Source: PLoS Comput Biol. 2011 Jul 21;7(7):e1002099. doi: 10.1371/journal.pcbi.1002099 (PMC3140962; doi:10.1371/journal.pcbi.1002099)
Supplement: Figure S14 — Distances between chosen hydrogen atoms of paromomycin, compared with values from an NMR study of neomycin, which differs from paromomycin only with one chemical group, having OH instead of NH . The experimental values are taken from Table 1 in [Asensio, J. L. et al., Chemistry. 2002, 8, 5228–40] and are as follows: 1 – H1Glc–H4Strp, 2 – H1Glc–H5Strp, 3 – H1Glc–H3Strp, 4 – H1Glc–H5Rib,5 – H1Glc–H2Rib, 6 – H1Glc–H3Rib, 7 – H1Rib–H5Strp, 8 – H1Rib–H4Strp, 9 – H1Rib–H6Strp,10 – H2Rib–H6Strp, 11 – H1Rib–H4Rib, 12 – H1Ido–H3Rib, 13 – H1Ido–H2Rib, 14 – H1Ido–H4Rib. The asterisks (*) mark the distances that were described as larger than in the experimental work. (PDF) [file pcbi.1002099.s015.pdf]

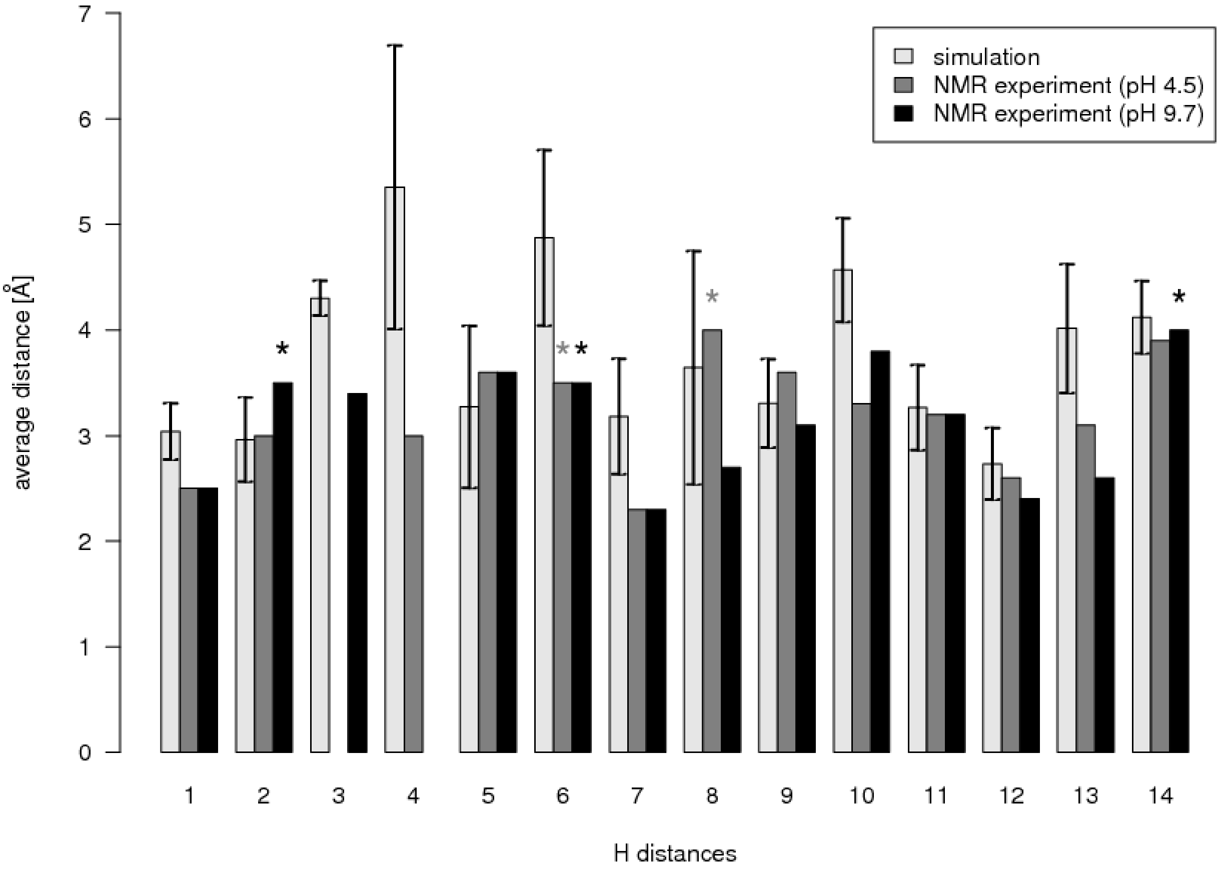

Figure S14: **Distances between chosen hydrogen atoms of paromomycin,** compared with values from an NMR study of neomycin, which differs from paromomycin only with one chemical group, having OH instead of NH_3^+. The experimental values are taken from Table 1 in [Asensio, J. L. *et al.*, Chemistry. **2002**, 8, 5228-40] and are as follows: 1 — $H1_{Glc}$–$H4_{Strp}$, 2 — $H1_{Glc}$–$H5_{Strp}$, 3 — $H1_{Glc}$–$H3_{Strp}$, 4 — $H1_{Glc}$–$H5_{Rib}$, 5 — $H1_{Glc}$–$H2_{Rib}$, 6 — $H1_{Glc}$–$H3_{Rib}$, 7 — $H1_{Rib}$–$H5_{Strp}$, 8 — $H1_{Rib}$–$H4_{Strp}$, 9 — $H1_{Rib}$–$H6_{Strp}$, 10 — $H2_{Rib}$–$H6_{Strp}$, 11 — $H1_{Rib}$–$H4_{Rib}$, 12 — $H1_{Ido}$–$H3_{Rib}$, 13 — $H1_{Ido}$–$H2_{Rib}$, 14 — $H1_{Ido}$–$H4_{Rib}$. The asterisks (*) mark the distances that were described as “larger than” in the experimental work.
