# Supplementary material for: Understanding the Origins of Bacterial Resistance to Aminoglycosides through Molecular Dynamics Mutational Study of the Ribosomal A-Site
Source: PLoS Comput Biol. 2011 Jul 21;7(7):e1002099. doi: 10.1371/journal.pcbi.1002099 (PMC3140962; doi:10.1371/journal.pcbi.1002099)
Supplement: Table S1 — Distribution of the MD conformations into clusters. Occupancy of each of the clusters derived from different MD simulations; the cluster with the highest population (occupancy) is underlined. (PDF) [file pcbi.1002099.s016.pdf]

Table S1: **Distribution of the MD conformations into clusters.**

cluster no. →	**1**	**2**	**3**	**4**	**5**
**NON_MUT**	0.158	0.556	0.042	0.069	0.176
**NON_MUT_PAR**	0.474	0.341	0.133	0.045	0.007
**G1491A**	0.179	0.327	0.316	0.032	0.146
**G1491A_PAR**	0.044	0.106	0.299	0.525	0.027
**G1491U**	0.108	0.354	0.280	0.240	0.019
**G1491U_PAR**	0.413	0.204	0.023	0.183	0.177
**U1495C**	0.071	0.515	0.291	0.026	0.097
**U1495C_PAR**	0.120	0.149	0.399	0.222	0.110
**U1406C/U1495A**	0.244	0.449	0.148	0.073	0.086
**U1406C/U1495A_PAR**	0.289	0.028	0.066	0.166	0.451

Occupancy of each of the clusters derived from different MD simulations; the cluster with the highest population (occupancy) is underlined.
