# Supplementary material for: Understanding the Origins of Bacterial Resistance to Aminoglycosides through Molecular Dynamics Mutational Study of the Ribosomal A-Site
Source: PLoS Comput Biol. 2011 Jul 21;7(7):e1002099. doi: 10.1371/journal.pcbi.1002099 (PMC3140962; doi:10.1371/journal.pcbi.1002099)
Supplement: Table S2 — Base pairing. Percentage of simulation time when base pairs were formed (i.e., at least one hydrogen bond was present). Two values are shown for each simulation corresponding to two A-sites. data from our previous study (Romanowska J., Setny P., Trylska J., J. Phys. Chem. B 2008); this base is U in G1491A, G1491U, NON_MUT and U1495C; and C in U1406C/U1495A simulation; this base is U in G1491A, G1491U and NON_MUT; C in U1495C; and A in U1406C/U1495A simulation; this base is A in G1491A, and U in G1491U. (PDF) [file pcbi.1002099.s017.pdf]

Table S2: **Base pairing**

base pairs ↓		*simulation*									
		**G1491A**		**G1491U**		**U1495C**		**U1406C/U1495A**		**NON_MUT**[1]	
1406:[2]	1495[3]	99.53	97.81	97.85	98.99	80.62	84.16	89.40	89.63	96.38	97.01
A1408:	A1492	5.56	0.76	3.70	1.85	—	14.99	1.73	—	—	8.98
	A1493	48.68	35.73	47.28	25.44	6.11	23.96	—	—	49.75	24.99
C1409:	1491[4]	28.60	34.47	15.94	20.65	*not applicable*					
	A1492	32.70	15.96	18.32	9.65	*not applicable*					

Percentage of simulation time when base pairs were formed (i.e., at least one hydrogen bond was present). Two values are shown for each simulation corresponding to two A-sites.

[1] data from our previous study (Romanowska J., Setny P., Trylska J., *J. Phys. Chem. B* **2008**);
[2] this base is U in G1491A, G1491U, NON_MUT and U1495C; and C in U1406C/U1495A simulation;
[3] this base is U in G1491A, G1491U and NON_MUT; C in U1495C; and A in U1406C/U1495A simulation;
[4] this base is A in G1491A, and U in G1491U.
