# Supplementary material for: Understanding the Origins of Bacterial Resistance to Aminoglycosides through Molecular Dynamics Mutational Study of the Ribosomal A-Site
Source: PLoS Comput Biol. 2011 Jul 21;7(7):e1002099. doi: 10.1371/journal.pcbi.1002099 (PMC3140962; doi:10.1371/journal.pcbi.1002099)
Supplement: Table S4 — Reproduction of the crystal water molecules in simulations of the complexed A-site. Selected water molecules of the 1J7T crystal structure and the corresponding water density areas in the MD simulations with paromomycin. “” denotes water density areas higher than 0.22 water molecules per observed in the position of the corresponding crystallographic water molecule; “” denotes lack of high water density in this position. Brackets denote analogous water molecules located in the symmetric part of the RNA fragment. (PDF) [file pcbi.1002099.s019.pdf]

Table S4: **Reproduction of the crystal water molecules in simulations of the complexed A-site.**

water molecule ↓	**G1491U_PAR**	**G1491A_PAR**	**U1406C/U1495A_PAR**	**U1495C_PAR**	**NON_MUT_PAR**
W1	+	−	+	+	+
W4	+	+	+	+	+
(W7)	(−)	(−)	(−)	(−)	(−)
W8 (W54)	+(−)	−(+)	+(−)	−(−)	+(−)
W9 (W2)	+(+)	−(+)	−(+)	+(+)	+(+)
W12	−	−	−	−	−
W13	+	−	−	−	+
W14	+	+	+	+	+
W15	−	−	−	−	−
W19	−	−	−	−	+
W20	−	+	−	+	+
W25	+	+	+	+	+
W27	+	−	−	−	−
W28	+	−	−	+	−
W32	+	+	−	+	+
W44	+	+	−	+	+
(W45)	(−)	(−)	(−)	(−)	(−)
W49	+	+	+*(near)*	+	+
W51	−	+	+	+	−

Selected water molecules of the 1J7T crystal structure and the corresponding water density areas in the MD simulations with paromomycin. "+" denotes water density areas higher than 0.22 water molecules per $Å^3$ observed in the position of the corresponding crystallographic water molecule; "−" denotes lack of high water density in this position. Brackets denote analogous water molecules located in the symmetric part of the RNA fragment.
